# Transport engineering for improving the production and secretion of valuable alkaloids in *Escherichia coli*

**DOI:** 10.1016/j.mec.2021.e00184

**Published:** 2021-09-14

**Authors:** Yasuyuki Yamada, Miya Urui, Hidehiro Oki, Kai Inoue, Haruyuki Matsui, Yoshito Ikeda, Akira Nakagawa, Fumihiko Sato, Hiromichi Minami, Nobukazu Shitan

**Affiliations:** a Laboratory of Medicinal Cell Biology, Kobe Pharmaceutical University, Motoyamakita-machi, Higashinada-ku, Kobe, 658-8558, Japan; b Research Institute for Bioresources and Biotechnology, Ishikawa Prefectural University, Nonoichi-machi, Ishikawa, 921-8836, Japan; c Department of Plant Gene and Totipotency, Division of Integrated Life Science, Graduate School of Biostudies, Kyoto University, Kyoto, 606-8502, Japan; d Graduate School of Science, Osaka Prefecture University, Sakai, 599-8531, Japan

**Keywords:** Alkaloids, Metabolic engineering, Transport engineering, *E. coli*, Reticuline, AtDTX1

## Abstract

Microorganisms can be metabolically engineered to produce specialized plant metabolites. However, these methods are limited by low productivity and intracellular accumulation of metabolites. We sought to use transport engineering for producing reticuline, an important intermediate in the alkaloid biosynthetic pathway. In this study, we established a reticuline-producing *Escherichia coli* strain into which the multidrug and toxic compound extrusion transporter *Arabidopsis* AtDTX1 was introduced. AtDTX1 was selected due to its suitable expression in *E. coli* and its reticuline-transport activity. Expression of AtDTX1 enhanced reticuline production by 11-fold, and the produced reticuline was secreted into the medium. AtDTX1 expression also conferred high plasmid stability and resulted in upregulation or downregulation of several genes associated with biological processes, including metabolic pathways for reticuline biosynthesis, leading to the production and secretion of high levels of reticuline. The successful employment of a transporter for alkaloid production suggests that the proposed transport engineering approach may improve the biosynthesis of specialized metabolites *via* metabolic engineering.

## Highlights

• The transporter AtDTX1 was introduced into a reticuline-producing *E. coli* strain.
• AtDTX1 significantly increased reticuline production and secretion.
• AtDTX1 conferred high plasmid stability.
• AtDTX1 expression affected regulation of multiple metabolic pathways.
• Transport engineering can enhance plant metabolite production.

## Abbreviations

5-Methyl-THF: 5-methyl-tetrahydrofolate
ABC: ATP-binding cassette
BH4: tetrahydrobiopterin
CFU: colony-forming units
DEGs: differentially expressed genes
E4P: d-erythrose 4-phosphate
FC: Fold-Change
FW: fresh weight
G3P: d-glyceraldehyde 3-phosphate
GO: Gene Ontology
KEGG: Kyoto Encyclopedia of Genes and Genomes
MATE: multidrug and toxic compound extrusion
MCS: multiple cloning site
NBDs: nucleotide binding domains
NPF: nitrate transporter 1/peptide transporter family
PBS: phosphate-buffered saline
PUP: purine permease
R5P: d-ribose 5-phosphate
SAM: *S*-adenosyl-l-methionine
TMDs: transmembrane domains
TMM: Trimmed Mean of M-values
UPLC-MS: ultra-performance liquid chromatography mass spectrometry

## 1 Introduction

Specialized plant secondary metabolites perform diverse functions owing to their varied chemical structures. Many of these metabolites have been used as medicines (Rischer et al., 2013). However, meeting the commercial demand for these metabolites can be difficult owing to their low concentrations in plant cells, the risk of overharvesting causing plant extinction, and costly methods of chemical synthesis. To circumvent these problems, biosynthetic enzymes used to generate these metabolites have been studied, and the genes that encode the corresponding enzymes have been isolated. Further progress in this field has enabled the production of useful compounds by introducing biosynthetic genes into microorganisms such as *Escherichia coli* and yeast by metabolic engineering (Diamond and Desgagne-Penix, 2016; Li et al., 2018; Minami, 2013). Some useful drugs produced in microorganisms include artemisinic acid, the precursor of the anti-malarial drug artemisinin (Ro et al., 2006); thebaine, an opiate (Galanie et al., 2015; Nakagawa et al., 2016); cannabinoid, a potential medicinal compound (Luo et al., 2019); and tropane alkaloids, which act as inhibitors of neurotransmitters (Srinivasan and Smolke, 2020).

Although microbial production of central and specialized metabolites is now possible, growth retardation and low productivity have been reported in certain cases, which are mainly attributed to the cytotoxicity of either the substrates or products, or negative feedback regulation of biosynthetic enzymes. For example, yeast cells expressing a plant prenyltransferase and producing a prenylated flavonoid exhibited decreased growth and production of the end product when the cells were exposed to a high concentration of naringenin, a flavonoid substrate (Sasaki et al., 2009). In another case, the expression of stress-responsive and multidrug transporter genes was highly upregulated in yeast cells stimulated to produce artemisinic acid (Ro et al., 2008). Furthermore, most metabolites accumulate in cells, which requires their extraction and purification from various endogenous cellular metabolites. Therefore, additional approaches for alleviating these problems are necessary.

One approach involves the use of transporters that remove metabolites from cells. The introduction of transporters in metabolite-producing microorganisms might increase production *via* elimination of negative feedback inhibition and enable efficient recovery from the medium. Several examples of successful transport engineering have been reported for central metabolites such as alcohols (Boyarskiy et al., 2016), alkanes (Chen et al., 2013), and fatty alcohols (Hu et al., 2018). However, for specialized metabolites—most of which are valuable as medicinal resources and toxic to microorganisms owing to their strong biological activities—reports regarding the use of efflux pumps are scarce, although a few studies report transport engineering for enhanced production of terpenoids such as zeaxanthin or amorphadiene, using microbial efflux transporters (Doshi et al., 2013; Zhang et al., 2016). This is probably due to the dearth of knowledge regarding plant transporters for specialized metabolites. However, some researchers have isolated transporters implicated in the transport of specialized metabolites (Supplementary Fig. S1) (de Brito Francisco and Martinoia, 2018; Gani et al., 2021; Lv et al., 2016; Shitan, 2016; Shitan et al., 2014a; Shitan and Yazaki, 2020), including: ATP-binding cassette (ABC) transporters, which use the energy obtained from ATP hydrolysis to transport substrates (Hwang et al., 2016); multidrug and toxic compound extrusion (MATE) transporters, which efflux substrates as proton antiporters (Takanashi et al., 2014; Upadhyay et al., 2019); nitrate transporter 1/peptide transporter family (NPF) members, which import substrates through proton symport (Jorgensen et al., 2017); and purine permeases (PUPs), which also import substrates through proton symport (Jelesko, 2012).

**Fig. 1:**
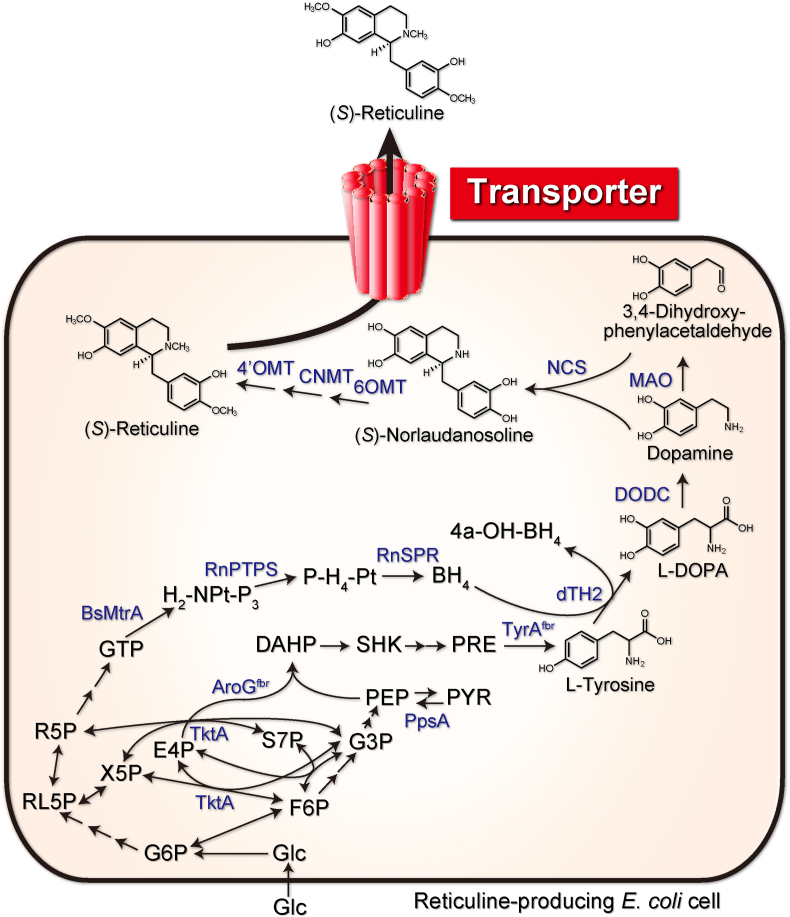
Schematic of the reticuline biosynthetic pathway engineered in *E. coli*. Reticuline is synthesized from simple carbon sources (i.e., glucose or sucrose) *via* the sequential action of enzymes dTH2, DODC, MAO, NCS, 6OMT, CNMT, and 4′OMT. As an efflux transporter of reticuline, AtDTX1 was introduced in this reticuline-producing *E. coli* strain. 4a-H-BH_4_, 4a-hydroxytetrahydrobiopterin; AroG^fbr^, 2-dehydro-3-deoxyphosphoheptonate aldolase; BH_4_, tetrahydrobiopterin; BsMtrA, GTP cyclohydrolase I; CNMT, coclaurine *N*-methyltransferase; dTH2, tyrosine hydroxylase; DAHP, 3-deoxy-d-arabino-heptulosonate-7-phosphate; DODC, l-DOPA decarboxylase; E4P, d-erythrose 4-phosphate; F6P, d-fructose 6-phosphate; G3P, d-glyceraldehyde 3-phosphate; G6P, d-glucose 6-phosphate; Glc, glucose; H_2_–NPt–P_3_, 7,8-dihydroneopterin triphosphate; MAO, monoamine oxidase; 4′OMT, 3′-hydroxy-*N*-methyl-(*S*)-coclaurine 4′-*O*-methyltransferase; 6OMT, norcoclaurine 6-*O*-methyltransferase; PEP, phosphoenolpyruvate; P–H_4_–Pt, 6-pyruvoyltetrahydropterin; PpsA, phosphoenolpyruvate synthetase; PRE, prephenate; PYR, pyruvate; R5P, d-ribose 5-phosphate; RL5P, d-ribulose 5-phosphate; RnPTPS, 6-pyruvoyltetrahydropterin synthase; RnSPR, sepiapterin reductase; S7P, d-sedoheptulose 7-phosphate; SHK, shikimate; TktA, transketolase; TyrA^fbr^, chorismate mutase-prephenate dehydrogenase (feedback-resistant); and X5P, d-xylose 5-phosphate.

Our group has been studying the transport mechanisms of alkaloids and has characterized the function of several transporters using microorganisms, including the ABCB-type of ABC transporters responsible for berberine translocation in *Coptis japonica* (Shitan et al., 2003, 2013), as well as four MATE transporters and one PUP transporter required for nicotine transport in *Nicotiana tabacum* (Kato et al., 2015; Morita et al., 2009; Shitan et al., 2014b; Shoji et al., 2009). In addition, other groups have reported several transporters of various alkaloids (de Brito Francisco and Martinoia, 2018; Shitan and Yazaki, 2020). Furthermore, using metabolic engineering, we have generated an *E. coli* strain that can produce reticuline (Matsumura et al., 2018), an important intermediate for various benzylisoquinoline alkaloids such as morphine and berberine *via* three engineered pathways, i.e., (1) an l-tyrosine-overproducing pathway *via* glycolysis, the pentose phosphate pathway, and the shikimic acid pathway; (2) a pathway producing dopamine from l-tyrosine along with the tetrahydrobiopterin (BH_4_)-synthesis pathway; and (3) a reticuline-producing pathway from dopamine (Fig. 1). Therefore, we hypothesized that the production and secretion of specialized metabolites such as reticuline can be enhanced by combining transport engineering with metabolic engineering in *E. coli* using the information available regarding alkaloid transporters.

In this study, we aimed to investigate the effect of introducing the MATE transporter AtDTX1 from *Arabidopsis thaliana*—selected due to its confirmed expression and transport activity in *E. coli*—on reticuline production and efflux in alkaloid-producing *E. coli*. Our results suggest that the combination of transport and metabolic engineering can be a powerful tool for enhancing the productivity of specialized plant metabolites in microorganisms. Broad application of this technology could lead to high production and stable supplies of useful pharmaceutical compounds in the future.

## 2 Materials and methods

### 2.1 Chemicals

(*S*)-reticuline was purified as described previously (Matsumura et al., 2018) and was used as a standard. The other chemicals used in this study were purchased from FujiFilm Wako Pure Chemical Corporation (Osaka, Japan) or Nacalai Tesque (Kyoto, Japan).

### 2.2 Construction of pCOLADuet-1_AtDTX1 and NtJAT1

Total RNA was isolated from *A. thaliana* seedlings (ecotype Columbia) using the RNeasy Plant Mini-kit (Qiagen, Hilden, Germany) per the manufacturer's instructions. The RNA was reverse-transcribed using SuperScript III reverse transcriptase (Invitrogen, Carlsbad, CA, USA), followed by incubation with RNaseH (Invitrogen). The full-length *AtDTX1* (At2g04040) sequence was amplified using reverse transcription-polymerase chain reaction (RT-PCR) from the cDNA. The primer sequences used are as follows:

AtDTX1_infusion2fw, 5′-ACCACAGCCAGGATCCGATGGAGGAGCCATTTCTTC−3′ and AtDTX1_infusion2rv, 5′-AAGCATTATGCGGCCGCTTAAGCCAATCTGTTTTCAGT-3′, where the underlined sequences indicate additional sequences for In-Fusion cloning. The PCR product was subcloned between the *Bam*HI and *Not*I sites of the pCOLADuet-1 (Novagen; Merck, Darmstadt, Germany) multiple cloning site (MCS)-1, which contains a 6 × His-tag for the creation of an N-terminal fusion using the In-Fusion HD cloning kit (Clontech, Mountain View, CA, USA).

The full-length *NtJAT1* sequence (accession no. AM991692) was amplified from the cDNA of *N. tabacum* using RT-PCR. The primer sequences used are as follows: NtJAT1_infusionfw, 5′-AAGGAGATATACATATGGTAGAGGAGTTGCCACAG-3′ and NtJAT1_infusionrv, 5′- TTACCAGACTCGAGGGTACCCTAAGATCTTCCTTCGTGTA-3′, where the underlined sequences indicate additional sequences for In-Fusion cloning. The PCR product was subcloned between the *Nde*I and *Kpn*I sites of the pCOLADuet-1 MCS-2 using the In-Fusion HD cloning kit. Protein expression was under the control of a T7*lac* promoter in pCOLADuet-1.

### 2.3 Expression of AtDTX1 and NtJAT1 in *E. coli*

BL21(DE3) cells harboring pCOLADuet-1, pCOLADuet-1_AtDTX1, or pCOLADuet-1_NtJAT1 were cultured overnight in liquid LB medium containing 50 mg/L kanamycin (Nacalai Tesque) at 30 °C with shaking at 200 rpm. Overnight cultures of OD_600_ = 0.1 were then inoculated in 90 mL LB medium containing 50 mg/L kanamycin and grown at 30 °C. Isopropyl β-D-thiogalactopyranoside (IPTG) (1 mM) was added when OD_600_ reached 0.6, then were incubated for another 3 h. Next, the cells were harvested *via* centrifugation at 9000×*g* for 2 min, re-suspended in 0.5 mL 1 × phosphate-buffered saline (PBS), and disrupted *via* sonication (nine cycles of 30 s on, 30 s off, performed on ice). The mixture was centrifuged at 3000×*g* for 15 min, and the supernatant was collected and centrifuged at 20,000×*g* for 20 min to pellet the membrane proteins. The membrane proteins were then denatured, subjected to electrophoresis on a 10% sodium dodecyl sulfate-polyacrylamide gel, and transferred to an Immobilon polyvinylidene difluoride membrane (Millipore, Tokyo, Japan). The membrane was treated with BlockingOne (Nacalai Tesque) and incubated with an anti-His monoclonal antibody (MBL Life Science, Nagoya, Japan) against His-AtDTX1 or an anti-NtJAT1 antibody (a generous gift from Dr. Y. Moriyama, Kurume University) against NtJAT1. The immunoreactive band was visualized using Chemi-Lumi One Super (Nacalai Tesque).

### 2.4 Reticuline transport by AtDTX1 and NtJAT1 in *E. coli* cells

BL21(DE3) cells harboring pCOLADuet-1, pCOLADuet-1_AtDTX1, or pCOLADuet-1_NtJAT1 were cultured as described in section 2.3. IPTG (1 mM) was added when OD_600_ reached 0.6, followed by incubation for another 3 h. The cells were harvested; suspended in LB containing 50 mg/L kanamycin, 1 mM IPTG, and 250 μM reticuline at OD_600_ = 0.7; and incubated at 30 °C while shaking at 200 rpm for 6 h. Next, the cells were harvested and washed with LB, and the reticuline content in the cells was quantified as described below.

### 2.5 Growth of *E. coli* BL21(DE3) cells expressing AtDTX1 or NtJAT1

*E. coli* BL21(DE3) cells harboring either pCOLADuet-1, pCOLADuet-1_AtDTX1, or pCOLADuet-1_NtJAT1 were cultured overnight in liquid LB containing kanamycin at 30 °C while shaking at 200 rpm. The overnight cultures were inoculated in 80 mL LB medium containing antibiotics as described above and grown at 25 °C. When OD_600_ reached approximately 0.6, each culture was divided into three 20 mL cultures in 100 mL baffled shake flasks and induced with 0.1 mM IPTG; the samples were further cultured at 25 °C with shaking at 200 rpm. Growth was measured by OD_600_.

### 2.6 Reticuline-producing *E. coli* cells

The genes encoding reticuline biosynthesis components were cloned in plasmids, as reported previously (Matsumura et al., 2018), with a different combination of genes and vectors (Table 1). These plasmids were introduced into *E. coli* BL21(DE3), yielding strain AN2104, which produces reticuline from a simple carbon source (Fig. 1). pCOLADuet-1, pCOLADuet-1_AtDTX1, or pCOLADuet-1_NtJAT1 were then introduced into AN2104.

**Table 1:** Plasmids used in this study.

Plasmids	Description	Source
pCOLADuet-1	Kanamycin resistance, expression vector	Novagen
pMW118tet	Tetracycline resistance, modified expression vector, pMW118	This study
pMW-TyrOE	*tyrA*^*fbr*^, *aroG*^*fbr*^, *tktA*, and *ppsA* in pMW118tet	This study
pCDF-MPSdTH2op	*BsMtrAop*, *RnPTPSop*, *RnSPRop*, and *dTH2op* in pCDFPL	Matsumura et al., (2018)
pET-NMop	*NCSop* and *MAOop* in pET-23a	Matsumura et al., (2018)
pACYC-3MT-DDC	*6OMT*, *CNMT*, *DODC*, and *4′OMT* in pACYC184	Matsumura et al., (2018)
pCOLA Duet-1 _AtDTX1	*AtDTX1* in pCOLADuet-1	This study
pCOLA Duet-1 _NtJAT1	*NtJAT1* in pCOLADuet-1	This study

op: codon optimized.

### 2.7 Growth of reticuline-producing, AtDTX1-expressing *E. coli*

Reticuline-producing *E. coli* harboring pCOLADuet-1 or pCOLADuet-1_AtDTX1 were cultured overnight in liquid LB containing 80 mg/L ampicillin (Sigma-Aldrich, St. Louis, MO, USA), 2 mg/L tetracycline (Nacalai Tesque), 100 mg/L spectinomycin (Nacalai Tesque), 30 mg/L chloramphenicol (Nacalai Tesque), and 50 mg/L kanamycin at 30 °C with shaking at 200 rpm. The overnight grown cultures were treated as described in section 2.5. Growth was measured by OD_600_.

### 2.8 Reticuline production

Reticuline-producing *E. coli* cells (AN2104 strain) harboring pCOLADuet-1 or pCOLADuet-1_AtDTX1 were cultured overnight in liquid LB containing antibiotics (see section 2.7) at 30 °C while shaking at 200 rpm. The overnight cultures were inoculated into 150 mL LB medium containing 9.4 g/L K_2_HPO_4_, 2.2 g/L KH_2_PO_4_, 0.4% glycerol, and antibiotics as described above, and grown at 30 °C. Each culture was divided into three 45 mL cultures in 300 mL baffled shake flasks when OD_600_ reached approximately 0.6, followed by the addition of 5 mL 30% glucose (3% final concentration) and IPTG (0.1 mM final concentration) for induction. The samples were further cultured at 25 °C while shaking at 150 rpm. The samples were harvested at 8, 12, 24, 48, and 72 h after induction.

### 2.9 UPLC-MS analysis for detection and quantification of reticuline

The cultured samples were separated into supernatants (medium) and pellets (cell) *via* centrifugation at 9000×*g* for 2 min. Trichloroacetate (2% final concentration) was added to the supernatants to precipitate the proteins, and then the supernatants were analyzed using an ACQUITY ultra-performance liquid chromatography mass spectrometry (UPLC-MS) system with a QDa mass detector (Waters Corp., Milford, MA, USA) after filtering using a 0.45 μm cosmospin filter (Nacalai Tesque). The pellets were incubated overnight with 15 μL/mg fresh weight (FW) methanol containing 0.1 N HCl. After centrifugation and filtration, the supernatants were also analyzed.

UPLC was performed using a CORTECS UPLC C18 column (2.1 × 100 mm, 1.6 μm; Waters Corp.) operated at 40 °C. The mobile phase A consisted of an aqueous solution of 0.01% acetic acid, while mobile phase B consisted of acetonitrile containing 0.01% acetic acid. Gradient elution was performed as follows: 0–9 min, 5%–40% B; 9–12 min, 40%–50% B; and 12–15 min, 50%–5% B. The flow rate and injection volume were set at 0.3 mL/min and 2 μL, respectively.

The QDa conditions were set as follows: cone voltage = 15 V, capillary voltage = 0.8 kV, and source temperature = 600 °C. Reticuline (m/z = 330) was detected using single-ion recording mode and identified by directly comparing its retention time and fragmentation spectrum (cone voltage = 50 V) with pure reticuline. The amount of reticuline was quantified using a standard curve.

### 2.10 RNA-seq analysis

Reticuline-producing *E. coli* cells were harvested at 0, 8, 12, 24, and 48 h after 0.1 mM IPTG treatment, and total RNA was extracted from *E. coli* cells using an RNeasy mini kit (Qiagen) after lysis with lysozyme (FujiFilm Wako) and proteinase K (Invitrogen). RNA sequencing and differentially expressed gene (DEG) analysis were carried out by Macrogen Japan Corp. (Kyoto, Japan) as follows: RNA sequencing with paired-end 101-bp reads was performed using a NovaSeq 6000 system (Illumina, San Diego, CA, USA). The quality check of the raw sequences and trimming were performed using FastQC (http://www.bioinformatics.babraham.ac.uk/projects/fastqc/) and Trimmomatic 0.38 (http://www.usadellab.org/cms/?page=trimmomatic), respectively. After read mapping using GCF_000022665.1_ASM2266v1 as a reference genome and Bowtie 1.1.2 (http://bowtie-bio.sourceforge.net/index.shtml), expression profiling was performed using HTSeq version 0.10.0 (http://www-huber.embl.de/users/anders/HTSeq/doc/overview.html). DEG analysis was performed on comparison pairs (e.g., AtDTX1 vs. VC1) using edgeR per the following workflow: 1) the read count value of known genes obtained through the HTseq were used as the original raw data. 2) Low-quality transcripts were filtered and Trimmed Mean of M-values (TMM) normalization was performed during data preprocessing. 3) Statistical analysis was performed using Fold-Change (FC), and exactTest with edgeR per comparison pair. Significance was accepted when |FC| ≥ 2 and exactTest raw *P*-value < 0.05. KEGG pathway mapping was carried out using KEGG Mapper (https://www.genome.jp/kegg/mapper.html). Hierarchical clustering and heatmap generation were performed using R version 3.4.4 (RCoreTeam, 2018).

### 2.11 Plasmid stability

Cell viability was estimated by counting the number of colony-forming units (CFU). Reticuline-producing *E. coli* cultures at 0, 4, and 24 h after 0.1 mM IPTG treatment were diluted 10,000-fold, 100,000-fold, and 1,000,000-fold, respectively, followed by plating 50 μL of the diluted samples on either antibiotic-free LB plates or plates containing five different antibiotics. CFU were determined after 24–36 h of growth at 25 °C. Plasmid stability was calculated by comparing the CFU between antibiotic-free and -supplemented plates.

### 2.12 Statistical analysis

Student's *t*-test (two-tailed) was used to determine significant differences compared to the control cells in reticuline production and plasmid stability analyses. Multiple comparisons were conducted using repeated analysis of variance (ANOVA) with the Bonferroni test for reticuline transport. Statistical significance was set at *P* < 0.05.

## 3 Results

### 3.1 Selection of appropriate transporters for transport engineering

To use transport engineering for alkaloid production, appropriate transporters involved in transporting specialized metabolites must be selected. First, we focused on the MATE family of plant transporters, which are known to transport specialized metabolites, efflux substrates from the cytosol (Supplementary Fig. S1), and have well-studied microbial expression. Among these, we focused on AtDTX1 from *A. thaliana* and NtJAT1 from *N. tabacum* (Supplementary Fig. S2). AtDTX1 transports plant-derived alkaloids such as berberine and palmatine with chemical structures relatively similar to those of reticuline (Supplementary Fig. S3). AtDTX1 was isolated from a functional screen using *E. coli* (Li et al., 2002), also confirming its expression in *E. coli*. On the other hand, NtJAT1 is well-expressed in *Saccharomyces cerevisiae* and localizes to the plasma membrane, where it shows substrate specificity for alkaloids such as berberine (Supplementary Fig. S2) (Morita et al., 2009). Therefore, we investigated the expression and reticuline transport activities of AtDTX1 and NtJAT1 in *E. coli* BL21(DE3).

**Fig. 2:**
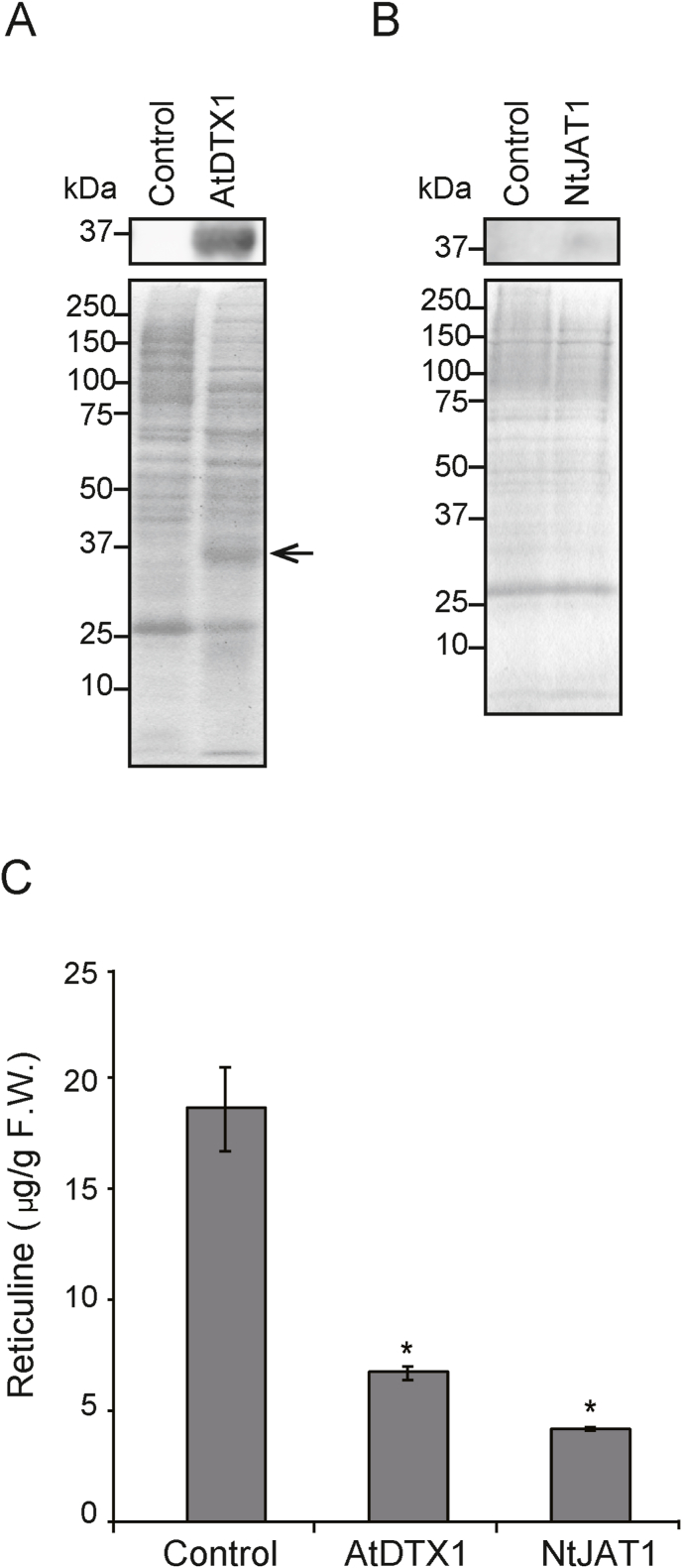
Reticuline transport activity of AtDTX1 and NtJAT1 in *E. coli* BL21(DE3). Expression of (A) AtDTX1 and (B) NtJAT1 was induced with 1 mM isopropyl β-D-thiogalactopyranoside (IPTG) and incubating for 3.5 h. Membrane proteins (10 μg per lane) of *E. coli* expressing AtDTX1, NtJAT1, or vector control were extracted, separated *via* sodium dodecyl sulfate-polyacrylamide gel electrophoresis, and blotted onto a polyvinylidene difluoride membrane. The membrane was probed with anti-His antibodies against AtDTX1 or anti-NtJAT1 antibodies against NtJAT1. The position of AtDTX1 is marked by an arrowhead. (C) Reticuline transport activity of MATE transporters. After induction of transporter proteins by adding 1 mM IPTG for 3 h, control, or AtDTX1-or NtJAT1-expressing *E. coli* cells were cultured in LB containing 250 μM reticuline for 6 h. Results are presented as the mean ± standard deviation of triplicates. Asterisks indicate a statistically significant difference compared to the control (ANOVA Bonferroni test; *P < 0.01). F.W., fresh weight.

Each cDNA was subcloned into pCOLADuet-1 and introduced to *E. coli* BL21(DE3). A 37 kDa immunoreactive band was detected only in the membrane preparation of *E. coli* transformed with *AtDTX1* cDNA using an anti-His antibody, which was confirmed by Coomassie Brilliant Blue staining (Fig. 2A), indicating that AtDTX1 was expressed in *E. coli*. In contrast, a weak band around 37 kDa was observed for NtJAT1 using the anti-NtJAT1 antibody, and its expression was not observed in Coomassie Brilliant Blue staining (Fig. 2B).

Next, the transformants were incubated in LB medium containing 250 μM reticuline, which enters the cells probably by diffusion. The intracellular reticuline content was quantitatively analyzed using UPLC-MS. Cells expressing AtDTX1 or NtJAT1 accumulated significantly less reticuline content than control cells (Fig. 2C). These data suggested that both MATE transporters were expressed at the plasma membrane of *E. coli* cells and effluxed reticuline.

During the reticuline transport assay, we observed that the growth of NtJAT1-expressing *E. coli* cells was significantly inhibited. As the expression of membrane proteins tends to reduce cell growth (Boyarskiy et al., 2016), the growth rate of *E. coli* cells expressing AtDTX1 or NtJAT1 was determined. In the presence of 0.1 mM IPTG, AtDTX1 expression in BL21(DE3) cells retarded growth slightly, whereas NtJAT1 expression significantly inhibited growth (Supplementary Fig. S4). NtJAT1 expression, which was not as high as AtDTX1 but was confirmed by immunoblot analysis (Fig. 2B), would have a negative effect on *E. coli* cells. As a growth defect is undesirable for alkaloid production, only AtDTX1 was used for further analysis.

### 3.2 Determination of the conditions for transporter expression and cell culture

We introduced AtDTX1 or the control vector in reticuline-producing cells that were established *via* the introduction of four vectors with 14 genes (Table 1) related to reticuline biosynthesis into the BL21(DE3) strain as described previously (Matsumura et al., 2018). Reticuline-producing cells grew more slowly than the parent BL21(DE3) strain, probably because these cells harbored multiple vectors and were cultured in the presence of antibiotics. AtDTX1 expression did not significantly affect the proliferation of *E. coli* cells in the presence of 0.1 mM IPTG (Supplementary Fig. S5). A previous report (Nakagawa et al., 2012) showed that reticuline is efficiently produced using a modified LB medium (pH 7.0) containing K_2_HPO_4_, KH_2_PO_4_, and glycerol (0.4%). Since AtDTX1 shows high activity at pH 7.0–8.0 (Li et al., 2002), this medium would be suitable for AtDTX1. Hence, we decided to investigate the reticuline production of these cells using this modified LB medium in the presence of 0.1 mM IPTG.

### 3.3 AtDTX1 significantly improved reticuline production and secretion to the medium

**Fig. 3:**
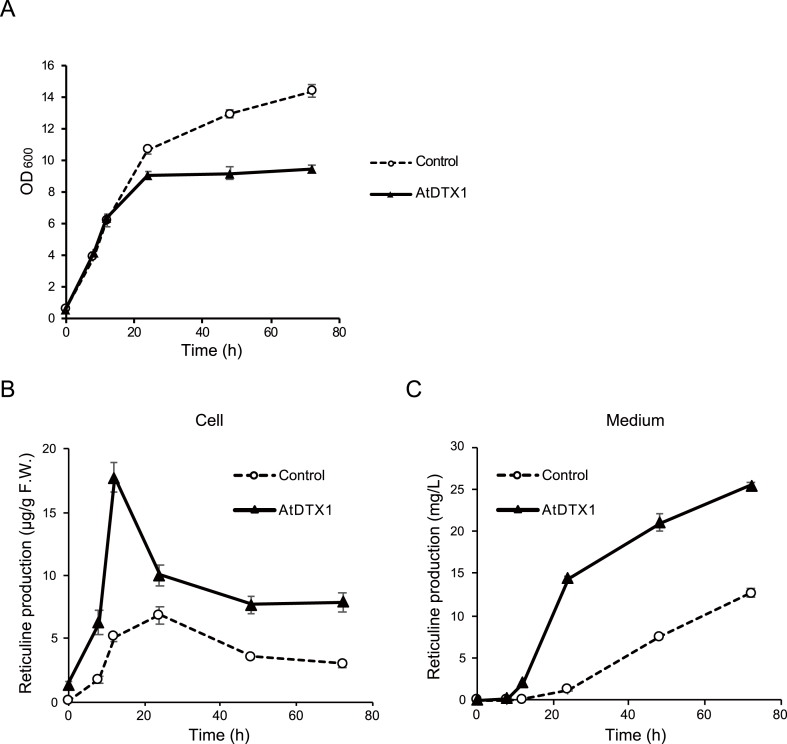
Reticuline production in *E. coli* cells and medium. (A) Growth was evaluated by measuring OD_600_, and the time-dependent production of (*S*)-reticuline in (B) *E. coli* cells and (C) the medium was determined. Control (dashed line) and AtDTX1-expressing (solid line) *E. coli* cells were cultured in modified LB medium containing 9.4 g/L K_2_HPO_4_, 2.2 g/L KH_2_PO_4_, 0.4% glycerol, and antibiotics. Isopropyl β-D-thiogalactopyranoside (IPTG) (0.1 mM final concentration) was added at OD_600_ = 0.6 (time = 0 h) and sampled at the time points indicated. Results are presented as the mean ± standard deviation of triplicates.

We next determined the effect of AtDTX1 expression on cell growth and reticuline production under the optimized conditions described above. At OD_600_ = 0.6 (time = 0 h), 0.1 mM IPTG was added to the medium to induce expression of reticuline biosynthetic enzymes and AtDTX1. AtDTX1-expressing cells grew in a similar manner to the control cells during exponential phase; however, they entered stationary phase earlier than the control cells (Fig. 3A). Reticuline levels in the cells and medium were quantitatively analyzed using UPLC-MS (Supplementary Fig. S6). Time-course analysis (from 0 h to 72 h) showed that the cellular reticuline content in AtDTX1-expressing *E. coli* cells was significantly higher than the empty vector control (Fig. 3B). At 0 h, slight reticuline production was observed, probably due to the leaky transcription of the Lac promoter (Penumetcha et al., 2010), and AtDTX1-expressing cells showed a slight increase in cellular reticuline compared to control. At 12 h, the cellular reticuline content in AtDTX1-expressing cells reached 17.7 μg/g FW, which was 3.5-fold higher than the control (5.1 μg/g FW). Reticuline was detected in the medium of AtDTX1-expressing cells (0.23 mg/L at 8 h) earlier than in the control medium (0.13 mg/L at 12 h). Reticuline content in the medium of AtDTX1-expressing cells sharply increased from 12 h to 24 h and, at 24 h, the difference in reticuline content between the two cell lines was 11-fold (14.4 mg/L in AtDTX1-expressing cells vs. 1.3 mg/L in control cells); this difference was maintained for 72 h (Fig. 3C). At 72 h, the difference in intracellular reticuline content was 2.6-fold (7.9 μg/g FW in AtDTX1-expressing cells vs. 3.0 μg/g FW in control cells) and the difference in medium reticuline content was 2.0-fold (25.5 mg/L in AtDTX1-expressing cells vs. 12.7 mg/L in control cells). These results indicated that AtDTX1 expression significantly enhanced reticuline production and secretion in the medium.

### 3.4 Plasmid stability and transcriptomic analysis of AtDTX1-expressing *E. coli* cells

**Fig. 4:**
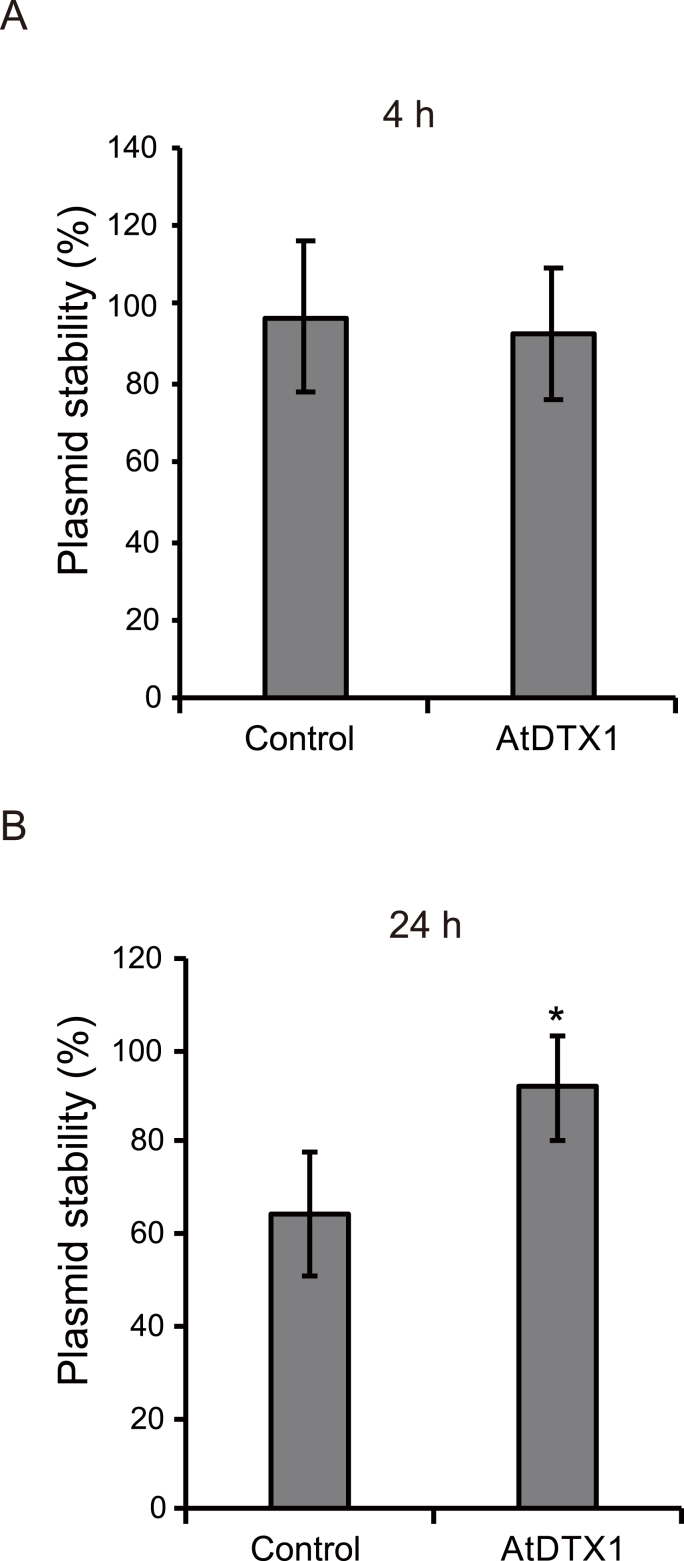
Plasmid stability of reticuline-producing *E. coli* cells. Stabilities of plasmids harboring either control vector or pCOLADuet-1_AtDTX1 grown in modified LB medium at (A) 4 h and (B) 24 h after adding 0.1 mM isopropyl β-D-thiogalactopyranoside (IPTG). Results are presented as the mean ± standard deviation (*n* = 9). Asterisks indicate a statistically significant difference compared to the control (Student's *t*-test; *P < 0.01).

A previous study demonstrated that metabolic engineering of yeast cells for the biosynthesis of artemisinic acid reduced plasmid stability and, therefore, artemisinic acid production (Ro et al., 2008). Thus, to understand the mechanism underlying the enhancement of reticuline production due to AtDTX1 expression, we assessed plasmid stability in reticuline-producing cells. At 4 h, both AtDTX1-expressing and control cells maintained similar plasmid numbers. However, at 24 h, 91.7% of AtDTX1-expressing cells maintained plasmids, which was significantly higher than the proportion of control cells (64.3%) (Fig. 4, Supplementary Fig. S7). These results suggest that AtDTX1-mediated reticuline efflux improved plasmid stability.

Higher reticuline production in AtDTX1-expressing cells (Fig. 3) suggested that AtDTX1 expression upregulated or downregulated metabolic genes, especially those for reticuline biosynthesis. Therefore, we next investigated transcriptomic changes in these cells. We performed RNA-seq (Supplementary Data 1) and analyzed DEGs using edgeR. Compared to the empty vector control cells at each time point (0, 8, 12, 24, and 48 h), 1393 genes were induced or suppressed in AtDTX1-expressing cells, with |FC| ≥ 2 and exactTest raw *P*-value < 0.05 (Supplementary Data 2–7). These genes exhibited varying expression levels relative to the control (Supplementary Fig. S8). The number of DEGs increased in a time-dependent manner. At 48 h, 352 genes were upregulated and 402 were downregulated (Supplementary Fig. S9A).

Gene Ontology (GO) enrichment analysis indicated enrichment of several subcategories of biological process, cellular process, and molecular function, and also revealed differences between the number of upregulated and downregulated genes (Supplementary Figs. S9B–D). A large number of genes were categorized into metabolic process (GO:0008152), cellular process (GO:0009987), response to stimulus (GO:0050896), localization (GO:0051179), membrane (GO:0016020), cell part (GO: 0044464), catalytic activity (GO:0003824), transport activity (GO:0005215), and binding (GO 0005488). Several genes categorized as detoxification (GO:0098754) and antioxidant activity (GO:0016209) were upregulated in AtDTX1-expressing cells at 12, 24, and 48 h, whereas many genes categorized as localization (GO:0051179), membrane (GO:0016020), protein-containing complex (GO:0032991), and transporter activity (GO:0005215) were downregulated (Supplementary Figs. S9B–D).

**Fig. 5:**
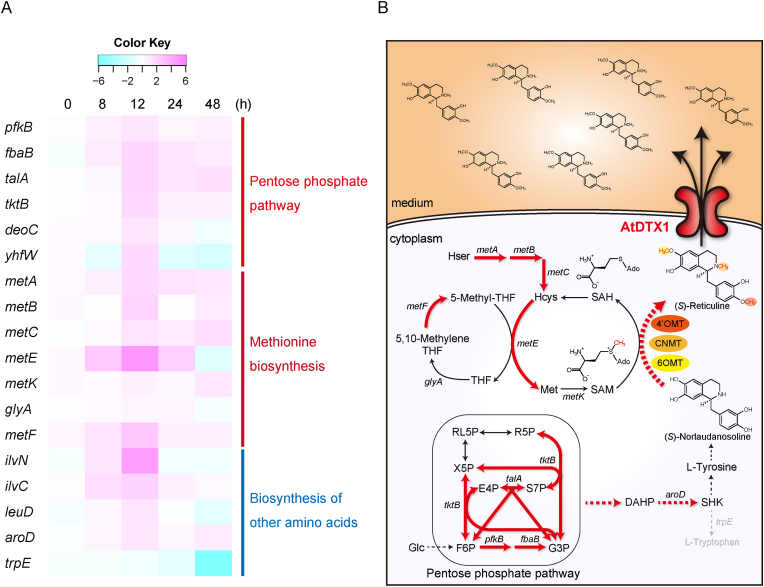
Summary of endogenous metabolic changes involved in reticuline biosynthesis. (A) Expression pattern of genes involved in the pentose phosphate pathway, methionine biosynthesis, and the biosynthesis of other amino acids. Heatmap generated with log2-based fold-change values showing the expression pattern. (B) Illustration of predicted metabolic changes involved in the improvement of reticuline production in AtDTX1-expressing *E. coli* cells. Red arrows indicate reaction steps involved in reticuline biosynthetic genes that were upregulated in AtDTX1-expressing cells with fold-change ≥2 at 12 h. Dotted lines represent multiple steps. *aroD*, 3-dehydroquinate dehydratase; *fbaB*, fructose-bisphosphate aldolase class 1; *glyA*, serine hydroxymethyltransferase; Hcys, homocysteine; Hser, homoserine; Met, methionine; *metA*, homoserine transsuccinylase; *metB*, cystathionine gamma-synthase; *metC*, cystathionine β-lyase; *metE*, cobalamin-independent homocysteine transmethylase; *metF*, 5,10-methylenetetrahydrofolate reductase; *metK*, methionine adenosyltransferase; *pfkB*, ATP-dependent 6-phosphofructokinase isozyme 2; SAH, *S*-adenosylhomocysteine; SAM, *S*-adenosylmethionine; *talA*, transaldolase A; *tktB*, transketolase 2; THF, tetrahydrofolate; and *trpE*, anthranilate synthase component 1.

In addition, we identified biological pathways regulated in AtDTX1-expressing cells by Kyoto Encyclopedia of Genes and Genomes (KEGG) analysis using the *E. coli* K-12 MG1655 pathway (Table 2, Supplementary Table S1 and Figs. S10–11). In “Global and overview maps,” the number of genes that were categorized into metabolic pathways (01100), biosynthesis of secondary metabolites (01110), microbial metabolism in diverse environments (01120), carbon metabolism (01200), biosynthesis of amino acids (01230), and biosynthesis of cofactors (01240) was high. In detailed categories, many genes in carbohydrate metabolism, energy metabolism, nucleotide metabolism, amino acid metabolism, and membrane transport were altered (Supplementary Table S1). Interestingly, at 12 h, the time of maximum difference in cellular reticuline production, several genes in the pentose phosphate pathway (00030) were upregulated (Fig. 5A, Supplementary Table S1 and Fig. S10). This pathway is involved in reticuline production by supplying various metabolites for reticuline, such as d-glyceraldehyde 3-phosphate (G3P), d-ribose 5-phosphate (R5P), and d-erythrose 4-phosphate (E4P) (Fig. 1, Fig. 5B). This induction may be caused by AtDTX1-dependent secretion of the end-product reticuline. Furthermore, methionine biosynthesis from homoserine was highly upregulated (Fig. 5A, Supplementary Fig. S10). The enhancement of methionine biosynthesis is also supported by the increased biosynthesis of 5-methyl-tetrahydrofolate (5-Methyl-THF), a methyl donor for methionine synthesis *via* homocysteine methyltransferase (Supplementary Fig. S10). Methionine is utilized for the biosynthesis of *S*-adenosyl-l-methionine (SAM), a methyl donor involved in reticuline biosynthesis *via* three methyl transferases, 4′OMT, CNMT, and 6OMT (Fig. 1, Fig. 5B). Some methyl transferases, such as 4′OMT and 6OMT, have been reported to be inhibited by end-products or related compounds, such as berberine or norreticuline (Morishige et al., 2000; Sato et al., 1994). Therefore, reticuline efflux from the cytosol *via* AtDTX1 may have relieved the negative feedback on methyltransferases, and methionine biosynthesis was enhanced to supply a sufficient amount of SAM. In addition to methionine, the biosynthesis of other amino acids such as valine, leucine, and isoleucine was upregulated at 12 h, and tryptophan biosynthesis was downregulated at 24 h, which might improve the production of reticuline (Fig. 5A, Supplementary Figs. S10–11). The expression changes of these endogenous genes were confirmed by quantitative real-time PCR, and almost similar profiles were observed (Supplementary Fig. S12). We also investigated the expression profiles of the introduced genes. Some genes such as OMT in AtDTX1-expressing cells showed lower expression than those in the control cells (Supplementary Fig. S13), however, the expression levels of all these exogenous genes (x10^10^) are much higher than that of the endogenous gene (x10^8^), suggesting that the corresponding enzymes would be sufficiently expressed.

**Table 2:** The number of genes upregulated or downregulated in KEGG pathways of “Global and overview maps” in AtDTX1-expressing cells.

Metabolism			Time point (h)				
		Reg.	0	8	12	24	48
Global and overview maps	01100 Metabolic pathways	Up	1	23	67	57	51
		Down	6	8	32	75	104
	01110 Biosynthesis of secondary metabolites	Up	0	9	32	18	15
		Down	3	0	13	36	45
	01120 Microbial metabolism in diverse environments	Up	0	6	19	10	12
		Down	2	5	14	26	25
	01200 Carbon metabolism	Up	0	7	15	4	5
		Down	0	0	7	8	8
	01210 2-Oxocarboxylic acid metabolism	Up	0	2	3	0	0
		Down	0	0	0	0	2
	01212 Fatty acid metabolism	Up	0	0	0	0	0
		Down	0	0	0	2	1
	01230 Biosynthesis of amino acids	Up	0	5	15	6	5
		Down	0	0	0	14	20
	01240 Biosynthesis of cofactors	Up	0	0	6	15	8
		Down	0	1	4	10	6
	01220 Degradation of aromatic compounds	Up	0	1	2	0	2
		Down	0	0	1	4	1

Reg, regulation.

These data suggested that AtDTX1 expression conferred high plasmid stability in the cells, significantly affected cellular processes, and induced biosynthetic flow in the cell (Fig. 5B). Overall, these changes led to the production of high amounts of reticuline and its efflux into the medium.

## 4 Discussion

Reports on the practical application of transporters for the production of specialized metabolites are scarce, as there is minimal information regarding plant transporters for specialized metabolites. In this study, we successfully utilized a transport engineering strategy for the efflux of plant specialized metabolites from an engineered *E. coli* strain. This study demonstrates the potential of using a plant alkaloid transporter for microbial production, with the product subsequently effluxed into the medium. Specifically, heterologous expression of an efflux transporter, AtDTX1, in reticuline-producing *E. coli* increased reticuline secretion by nearly 11-fold.

Selection of an appropriate transporter is important for transport engineering. In this study, we focused on MATE family transporters. This is because the NPF and PUP transporter import substrates through proton co-transport mechanisms, which is not appropriate for studying product efflux from the cytosol to the medium. Some plant ABC transporters show efflux activity, however, most plant ABC transporters of specialized metabolites function as a single polypeptide consisting of two transmembrane domains (TMDs) and two nucleotide binding domains (NBDs) (Hwang et al., 2016), which is quite different from prokaryotic ABC transporters (Orelle et al., 2019). Therefore, plant ABC transporters might not function efficiently in *E. coli*, and research into the functional analysis of plant ABCB transporters involved in alkaloid transport in *E. coli* is limited. In contrast, MATE transporters efflux substrates *via* a proton antiport mechanism, and the structure of plant MATE transporters is similar to that of microbial MATE transporters.

Plant MATE transporters efflux diverse substrates, including specialized metabolites, and their substrate specificities are related to sequence homology to some extent. In a phylogenetic tree (Supplementary Fig. S14), many transporters in clade I transport specialized metabolites such as: proanthocyanidin by AtTT12 and *Medicago truncatula* MATE1; anthocyanin by MtMATE2 and *Vitis vinifera* AM1; and alkaloids by NtJAT1 and CjMATE1 (Shitan and Yazaki, 2020; Takanashi et al., 2014). Transporters in clade II are implicated in plant development; however, the substrates of these transporters are not yet known, with the exception of AtDTX50, which transports abscisic acid (Upadhyay et al., 2019). Furthermore, AtEDS5 in clade III transports salicylic acid, whereas most clade IV transporters transport citrate and are involved in iron translocation or Al^3+^ detoxification (Takanashi et al., 2014).

Although many plant transporters of specialized metabolites have recently been identified (de Brito Francisco and Martinoia, 2018; Lv et al., 2016; Shitan, 2016), more information is required regarding their substrate specificities and expression in microorganisms. For utilizing plant transporters in microbial production, transporter expression and activity in the host microorganism should be assessed. In this study, we confirmed robust expression and reticuline-transport activity of AtDTX1 in *E. coli*. Interestingly, AtDTX1 increased secretion of biosynthetic intermediates, that is, (*S*)-norlaudanosoline and (*S*)-6-*O*-methylnorlaudanosoline (Supplementary Fig. S15). Since AtDTX1 transports berberine and palmatine, which are structurally similar to reticuline, these intermediates might be transported out. Using this relatively broad substrate specificity, this transporter would be utilized for transport engineering of other alkaloids with similar structures. In contrast, NtJAT1 expression was weak and hindered cell growth significantly, and further experimentation was hence ceased in this study. However, NtJAT1 is best suited for the transport engineering of yeast cells, as its expression and substrate specificity for alkaloids have been characterized in yeast cells (Morita et al., 2009).

The expression level and time required for the induction of the transporter should be carefully determined. Overexpression of the transporter, similar to other membrane proteins, inhibits cell growth (Boyarskiy et al., 2016). For example, *n*-butanol production using transport engineering required transporter expression optimization to balance its toxicity while ensuring efficient production (Boyarskiy et al., 2016). In this study, we suppressed the basal expression level of the transporter using a low copy plasmid, pCOLADuet-1. Nevertheless, significant growth inhibition was observed when expression was induced by high concentrations of IPTG (1 mM) at the lag phase of *E. coli* (OD_600_ = 0.1) (data not shown). Therefore, a low concentration of IPTG (0.1 mM) was added after the cells grew to some extent (OD_600_ = 0.6). This experimental condition negligibly affected cell growth in reticuline-producing cells, and still resulted in robust production and secretion of reticuline.

Several studies have reported using transport engineering for the production of central metabolites and some specialized metabolites such as codeine and tropane alkaloids (Dastmalchi et al., 2019; Srinivasan and Smolke, 2020); the transporters used for codeine and tropane alkaloids production play roles in the transport of biosynthetic intermediates from the medium to the cytosol or from the cytosol to the vacuolar lumen. However, these studies have mainly focused on the increase in production after transporter expression, and only a few studies have analyzed genetic changes in the engineered cells. In this study, we investigated intracellular alterations based on plasmid stability and transcriptomic analysis (Fig. 4, Fig. 5, Supplementary Figs. S7–11 and Data 1–7). The expression of several genes involved in metabolism, categorized into various metabolic pathways, biosynthesis of amino acids, and purine metabolism, were highly regulated. Interestingly, other cellular processes such as ribosome and ABC transporters were also highly regulated, suggesting diverse effects of AtDTX1 expression on cellular function. Notably, metabolic pathways related to reticuline biosynthesis were altered. In addition to the pentose phosphate pathway, the genes for the biosynthesis of several amino acids were also upregulated at 12 h post-induction. Downregulation of genes for tryptophan biosynthesis at 24 h might lead to higher production of tyrosine. AtDTX1-dependent reticuline efflux might cause the decrease of biosynthetic intermediates such as G3P, R5P, E4P, and tyrosine, along with SAM, the methyl donor for methyltransferases in reticuline biosynthesis, which possibly resulted in the upregulation of related cellular metabolism to compensate for the supply of these compounds. These alterations would have not only led to secretion to the medium but also higher production in the cells. These biosynthetic pathways are not only used for reticuline biosynthesis, but are also used for and involved in other metabolite pathways. Therefore, the alteration of gene expression in pentose phosphate pathways and others would have affected the gene expression of other pathways.

In contrast, AtDTX1 expression seems to induce stress in the cells. In the GO analysis, many genes categorized into response to stimulus, detoxification, and antioxidant activity were upregulated. As reticuline did not significantly inhibit the growth of *E. coli* (Supplementary Fig. S16), stress-related genes might be induced by AtDTX1 expression.

Surprisingly, plasmid stability in AtDTX1-expressing cells was high, suggesting that the percentage of reticuline-producing cells in the culture medium was also high. These findings indicate that the expression of AtDTX1 induced diverse changes in the host cells, and the integrated effect of these alterations increased production of the desired compound.

Application of metabolic engineering to efflux transporters enables strong production and efficient recovery of valuable specialized metabolites. Some synthesized metabolites accumulate *in vivo*; therefore, procedures for the extraction and purification of metabolites are necessary, which renders some microbial systems commercially unfeasible. Combining transport and metabolic engineering not only improves the production of valuable metabolites but is also beneficial for the efficient recovery of the products. Although knowledge about transporters for transport engineering is still limited, and the identification of optimal transporters for desired metabolites would be necessary, further progress in this field will enhance the production of medicinal resources from plants.

## 5 Conclusions

We successfully developed an *E. coli* transport engineering platform that enables production and secretion of a valuable alkaloid at high levels. The results of the present study are of considerable significance, as *E. coli* is a standard microorganism for industrial-scale production of plant medicines (Diamond and Desgagne-Penix, 2016; Minami, 2013; Nakagawa et al., 2016). The platform proposed here, which combines metabolic and transporter engineering will provide opportunities for the low-cost production of various valuable alkaloids. The use of this technology for rapid and mass production of useful plant metabolites will contribute to the health and welfare of society.

## Data availability statement

Data supporting the findings of this work are available within the paper and its Supplementary Information. RNA-seq data are available from the DDBJ Sequenced Read Archive under accession number DRA011247. All relevant data presented in this paper are available from the corresponding author upon reasonable request.

## Author statement

**Yasuyuki Yamada:** Conceptualization, Methodology, Software, Validation, Formal analysis, Investigation, Data Curation, Writing – Original Draft, Writing – Review & Editing, Visualization. **Miya Urui:** Investigation. **Hidehiro Oki:** Investigation. **Kai Inoue:** Investigation. **Haruyuki Matsui:** Investigation. **Yoshito Ikeda:** Investigation. **Akira Nakagawa:** Resources. **Fumihiko Sato:** Conceptualization, Methodology, Writing – Review & Editing. **Hiromichi Minami:** Conceptualization, Methodology, Resources, Writing – Review & Editing. **Nobukazu Shitan:** Conceptualization, Methodology, Software, Validation, Formal analysis, Investigation, Data Curation, Writing – Original Draft, Writing – Review & Editing, Visualization, Project administration, Funding acquisition.

## Declaration of competing interest

The authors declare that they have no known competing financial interests or personal relationships that could have appeared to influence the work reported in this paper.
