# Supplementary material for: Transport engineering for improving the production and secretion of valuable alkaloids in Escherichia coli
Source: Metab Eng Commun. 2021 Sep 14;13:e00184. doi: 10.1016/j.mec.2021.e00184 (PMC8449128; doi:10.1016/j.mec.2021.e00184)
Supplement: Multimedia component 8 [file mmc8.pdf]

Supplementary Information

Transport engineering for improving the production and secretion of valuable alkaloids in *Escherichia coli*

Yasuyuki Yamada[a], Miya Urui[a], Hidehiro Oki[a], Kai Inoue[a], Haruyuki Matsui[a], Yoshito Ikeda[a], Akira Nakagawa[b], Fumihiko Sato[c,d], Hiromichi Minami[b], Nobukazu Shitan[a*]

[a] Laboratory of Medicinal Cell Biology, Kobe Pharmaceutical University, Motoyamakita-machi, Higashinada-ku, Kobe 658-8558, Japan

[b] Research Institute for Bioresources and Biotechnology, Ishikawa Prefectural University, Nonoichi-machi, Ishikawa 921-8836, Japan

[c] Department of Plant Gene and Totipotency, Division of Integrated Life Science, Graduate School of Biostudies, Kyoto University, Kyoto 606-8502, Japan

[d] Graduate School of Science, Osaka Prefecture University, Sakai 599-8531, Japan

*Correspondence: Nobukazu Shitan

E-mail: shitan@kobepharma-u.ac.jp (NS)

Supplementary methods

Quantitative RT-PCR analysis

Total RNA was extracted from *E. coli* cells as described in the **Materials and Methods section 2.10**. Single-stranded cDNA was synthesized from 1 μg of total RNA with the ReverTra Ace qPCR RT Master Mix using the gDNA Remover Kit (TOYOBO, Osaka, Japan). Real-time PCR was performed with specific primer pairs using the THUNDERBIRD Next SYBR qPCR Mix (TOYOBO) on the LightCycler 96 system (Roche, Basel Switzerland). The PCR conditions were 95°C for 30 s, followed by 40 cycles of 95°C for 5 s and 60°C for 30 s. The mRNA expression levels of *E. coli* endogenous genes were quantified using a standard curve prepared from dilution series of pT7Blue plasmids harboring partial sequence of target genes. The mRNA expression levels of exogenous genes were also quantified using each plasmid listed in Table 1. Primers used are shown in Table S2.

UPLC-MS analysis for detection and quantification of intermediates

The medium samples were prepared and analyzed as described in 2.9. UPLC was performed using an ACQUITY UPLC BEH C18 Column (2.1 × 100 mm, 1.7 μm; Waters Corp.) operated at 30 °C. The mobile phase A consisted of an aqueous solution of 0.01% acetic acid, while mobile phase B consisted of acetonitrile containing 0.01% acetic acid. Gradient elution was performed as follows: 0–2 min, 2% B; 2–4 min, 2%–6% B; 4–14 min, 6%–35% B; 14–15 min, 35%–98% B; 15–17 min, 98% B; and 17–18 min, 98%–2% B; and 18–20 min, 2% B. The flow rate and injection volume were set at 0.3 mL/min and 2 μL, respectively.

The QDa conditions were set as follows: cone voltage = 20 V, capillary voltage = 0.8 kV, and source temperature = 600 °C. Norlaudanosoline (NLS) (m/z = 288) and 6-*O*-methylnorlaudanosoline (6-*O*-methyl NLS) (m/z = 302) were detected using single-ion recording mode and identified by directly comparing their retention time. Fragmentation spectrum (cone voltage = 45 V) with pure NLS was also confirmed. The amount of NLS and 6-*O*-methyl NLS was quantified using a standard curve.

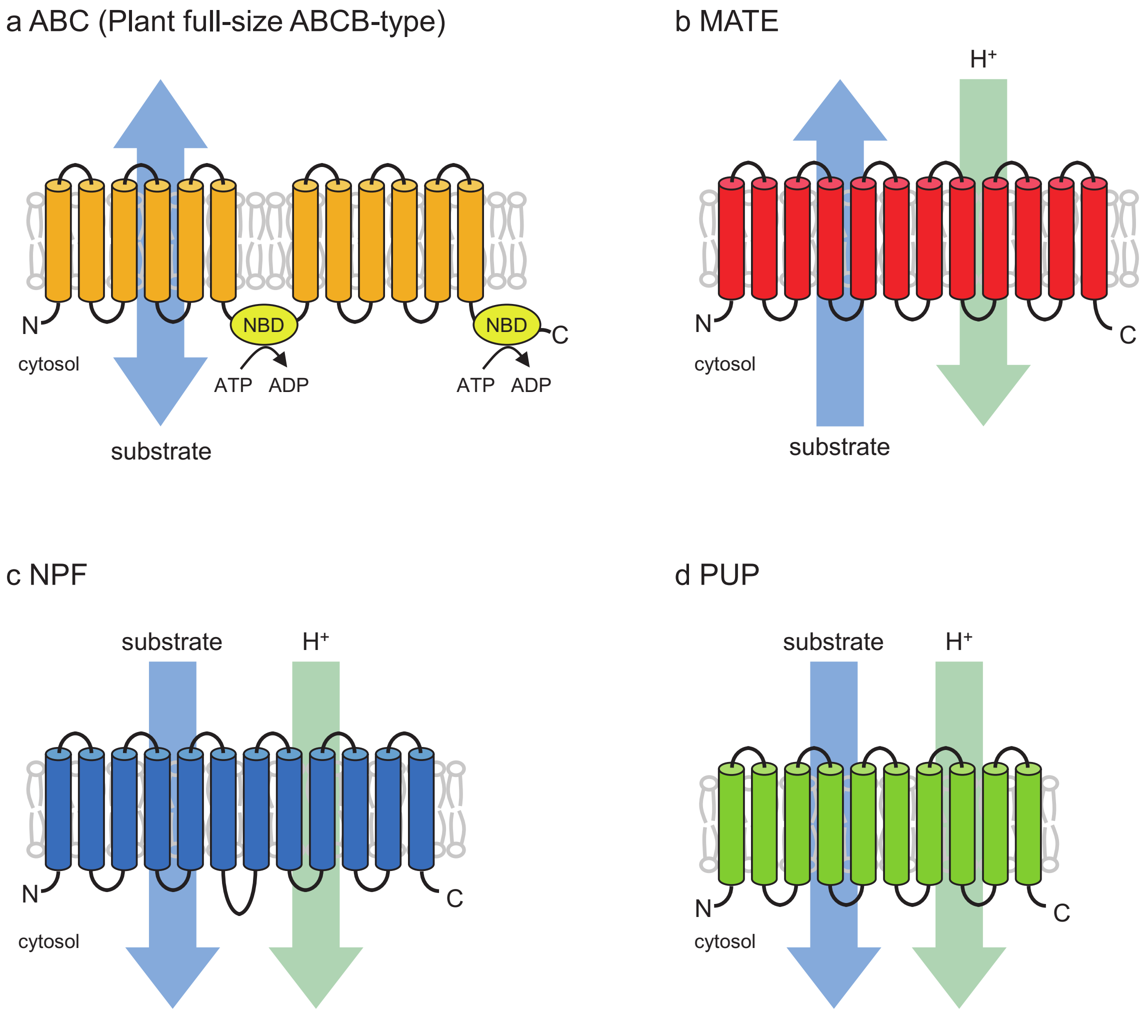

Supplementary Fig. S1 Representative structure of plant transporters involved in transporting specialized metabolites and the direction of transport. (a) ABC (plant full-size ABCB-type) transporter. (b) MATE transporter. (c) NPF transporter. (d) PUP transporter. ABC, ATP-binding cassette; MATE, multidrug and toxic compound extrusion; NPF, nitrate transporter 1/peptide transporter family; PUP, purine permease.

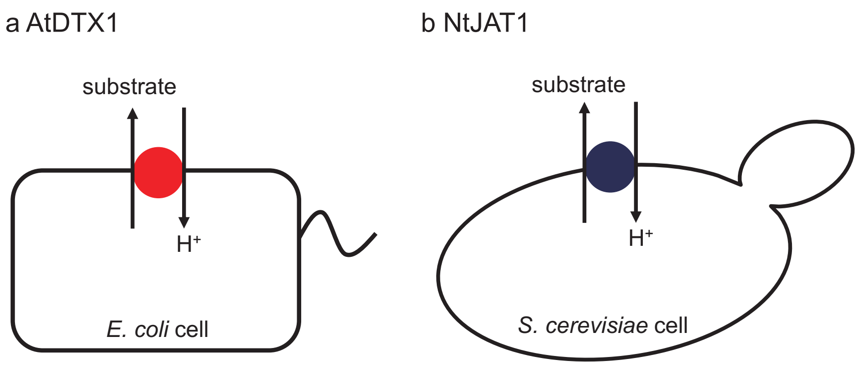

	AtDTX1	NtJAT1
Organism	*Arabidopsis thaliana*	*Nicotiana tabacum*
Subcellular localization	Plasma membrane	Vacuolar membrane (*N. tabacum*)<br>Plasma membrane (yeast cells)
Functional characterization	Heterologous expression and transport analysis using *E. coli* cells	Heterologous expression and transport analysis using yeast cells or insect cells (proteoliposeome assay)
Substrates	Berberine, palmatine, norfloxacin, ethidium bromide, Cd^{2+}	Nicotine, berberine, hyoscyamine, anabasine, rhodamine, ethidium bromide, verapamil
Reference	Li et al., *Journal of Biological Chemistry,* 277, 5360-5368 (2002)	Morita et al., *Proceedings of the National Academy of Sciences of the United States of America* 106, 2447-2452 (2009)

Supplementary Fig. S2 Model showing AtDTX1 (a) and NtJAT1 (b) function in microorganisms.

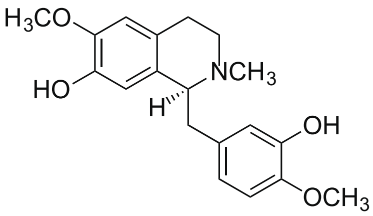

(*S*)-Reticuline

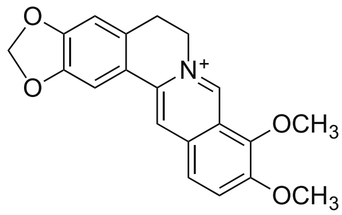

Berberine

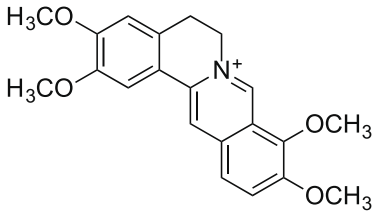

Palmatine

Supplementary Fig. S3 Structures of reticuline, berberine, and palmatine.

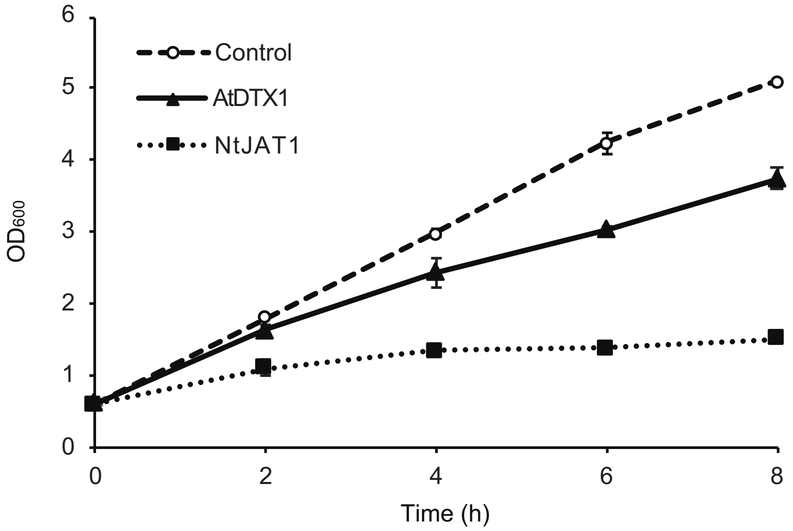

Supplementary Fig. S4 Growth of *E. coli* BL21(DE3) cells expressing AtDTX1 or NtJAT1. Growth was evaluated by measurement of the optical density at 600 nm.

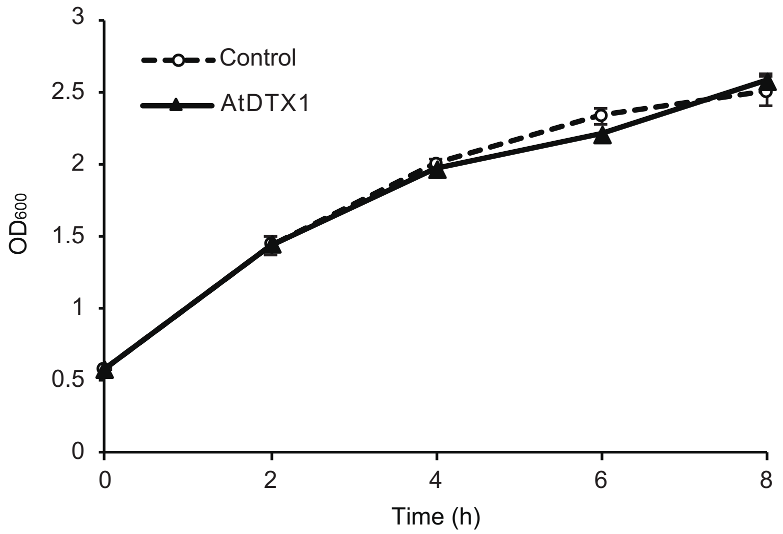

Supplementary Fig. S5 Growth of reticuline-producing *E. coli* expressing AtDTX1.

Growth was evaluated by measuring the optical density at 600 nm.

a Reticuline (authentic standard) b Reticuline (medium)

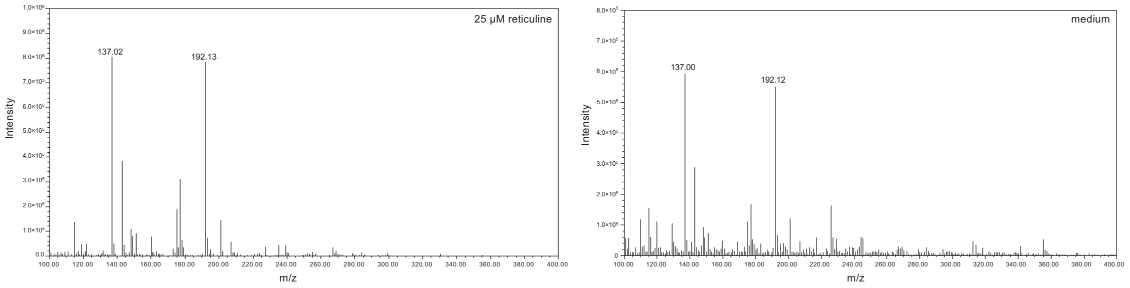

Supplementary Fig. S6 Fragmentation patterns of authentic reticuline (a) and reticuline extracted from medium (b).

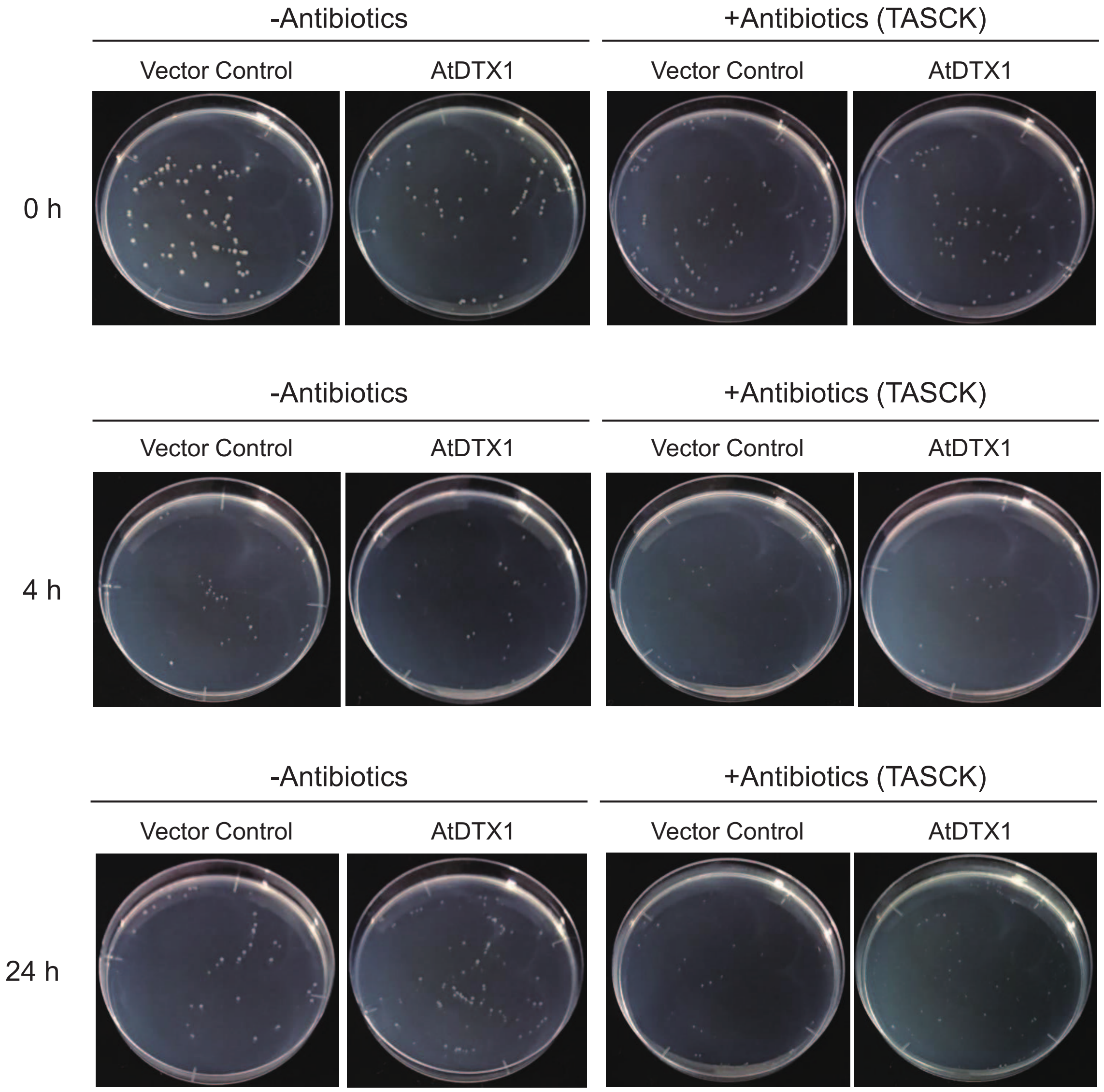

Supplementary Fig. S7 Representative image of colonies on LB medium with or without antibiotics. TASCK denotes tetracycline, ampicillin, spectinomycin, chloramphenicol, and kanamycin.

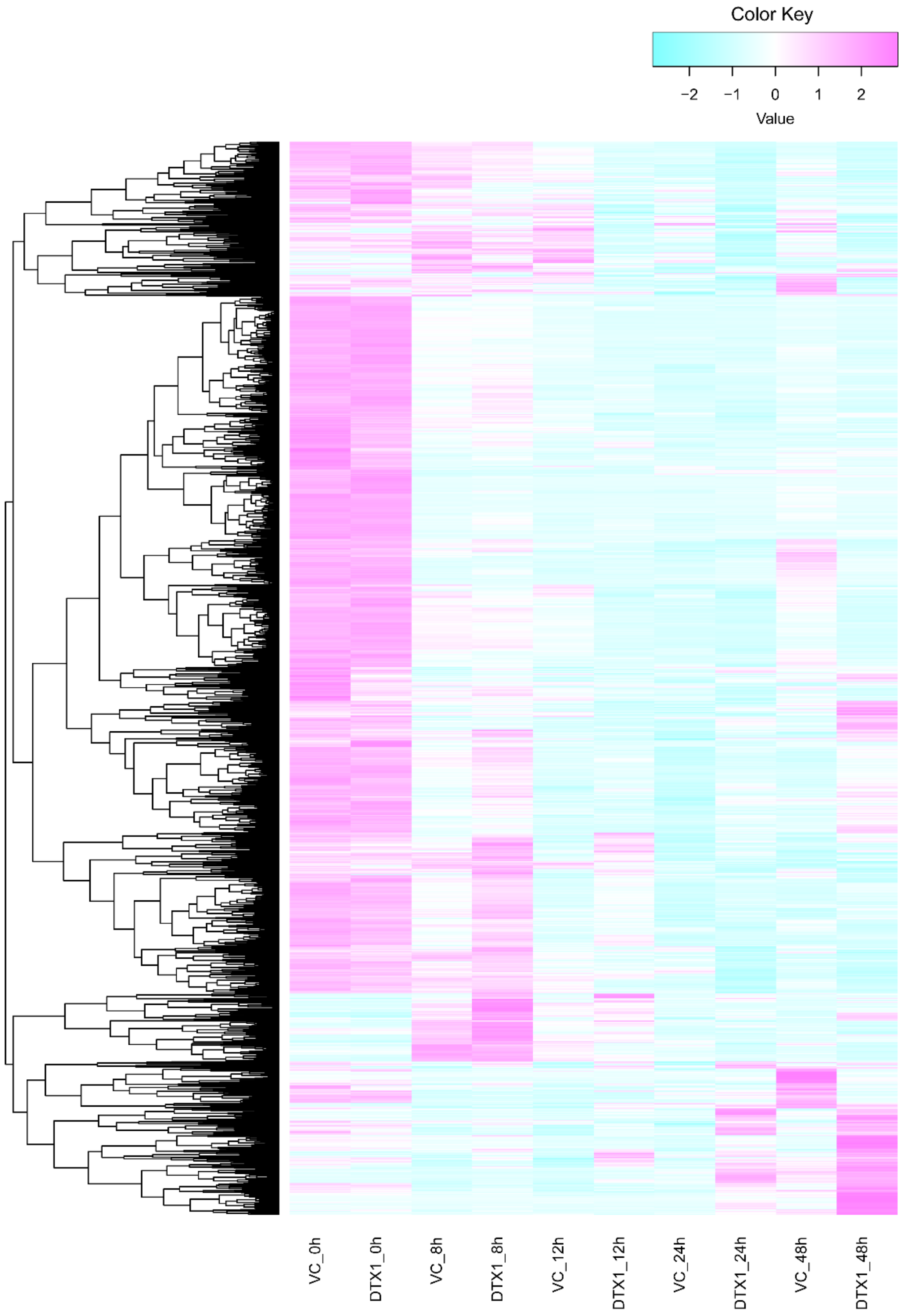

Supplementary Fig. S8 Heatmap of 1,393 genes upregulated or downregulated in AtDTX1-expressing cells, with |fold change| ≥ 2. Red indicates upregulation and blue indicates downregulation of each gene.

A Total genes

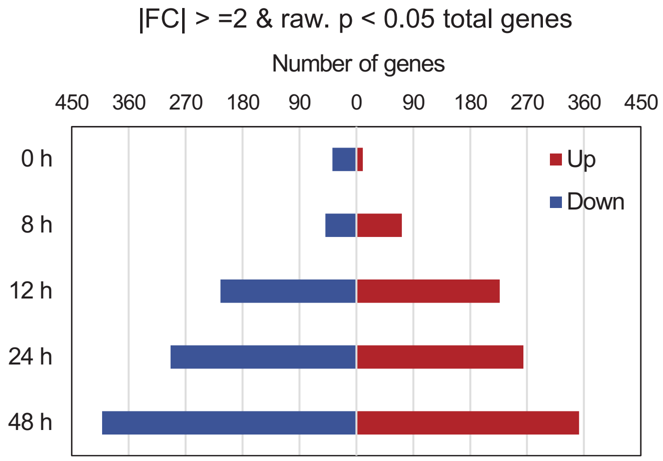

B Biological process

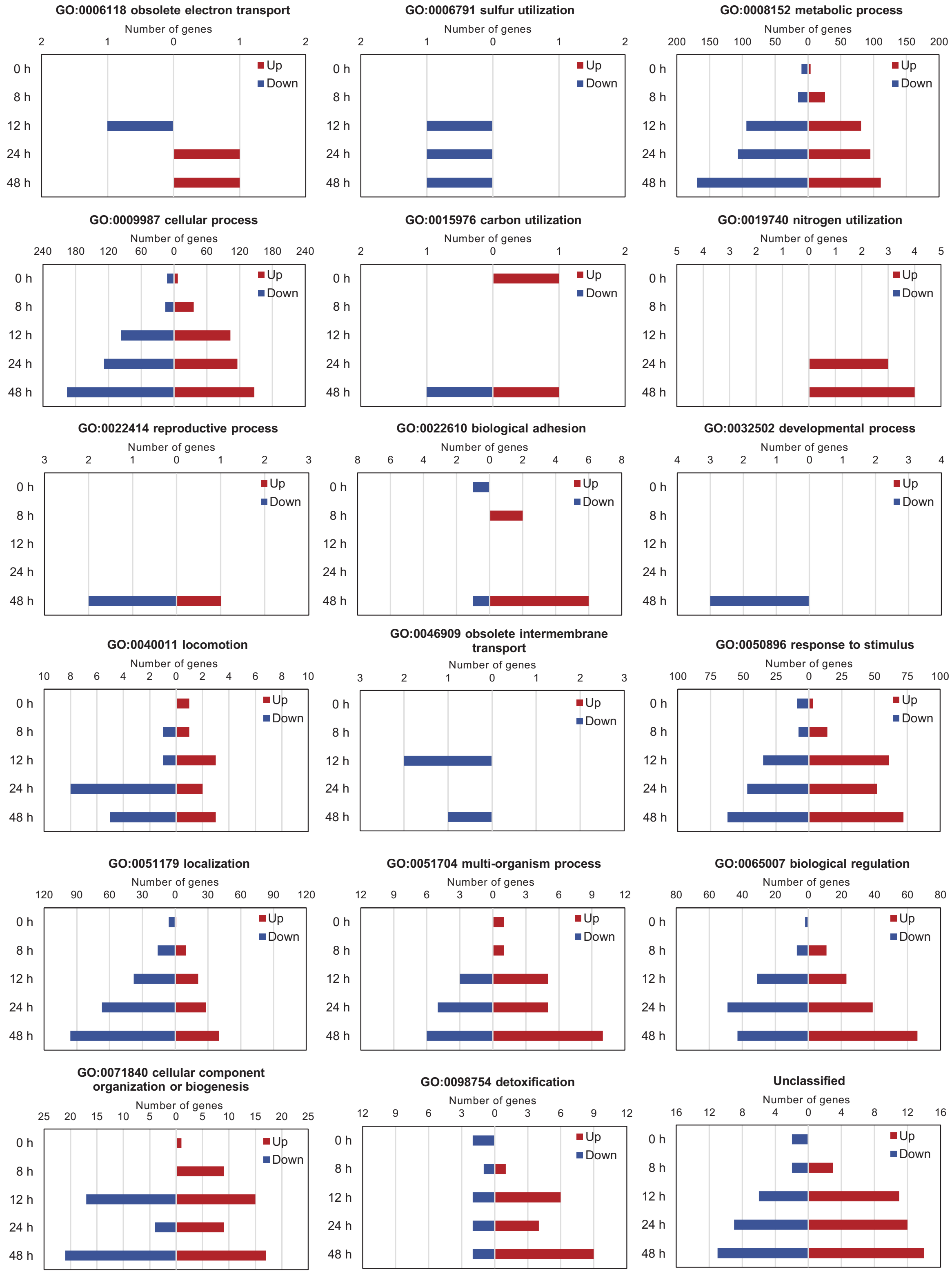

C Cellular process

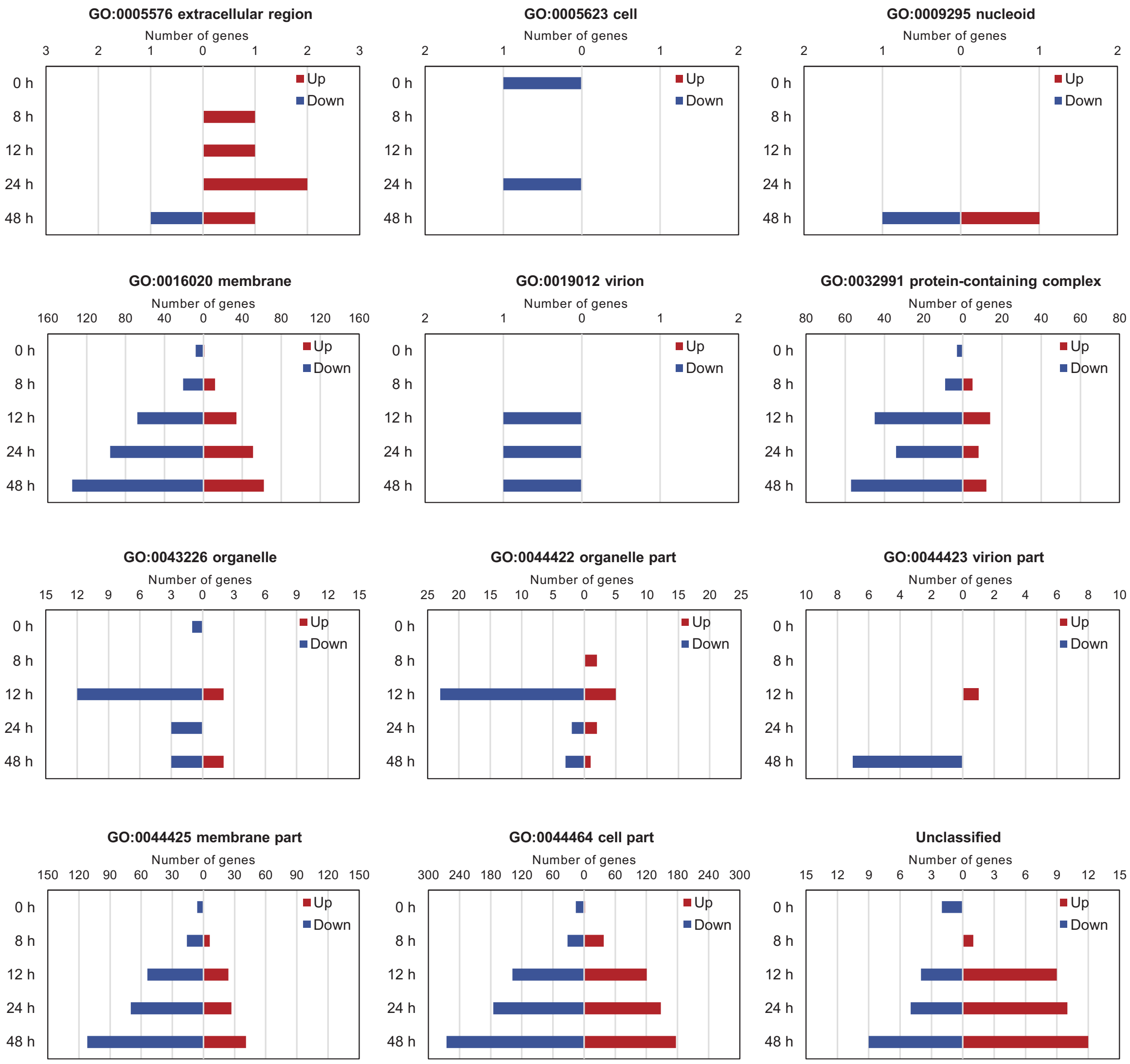

D Molecular function

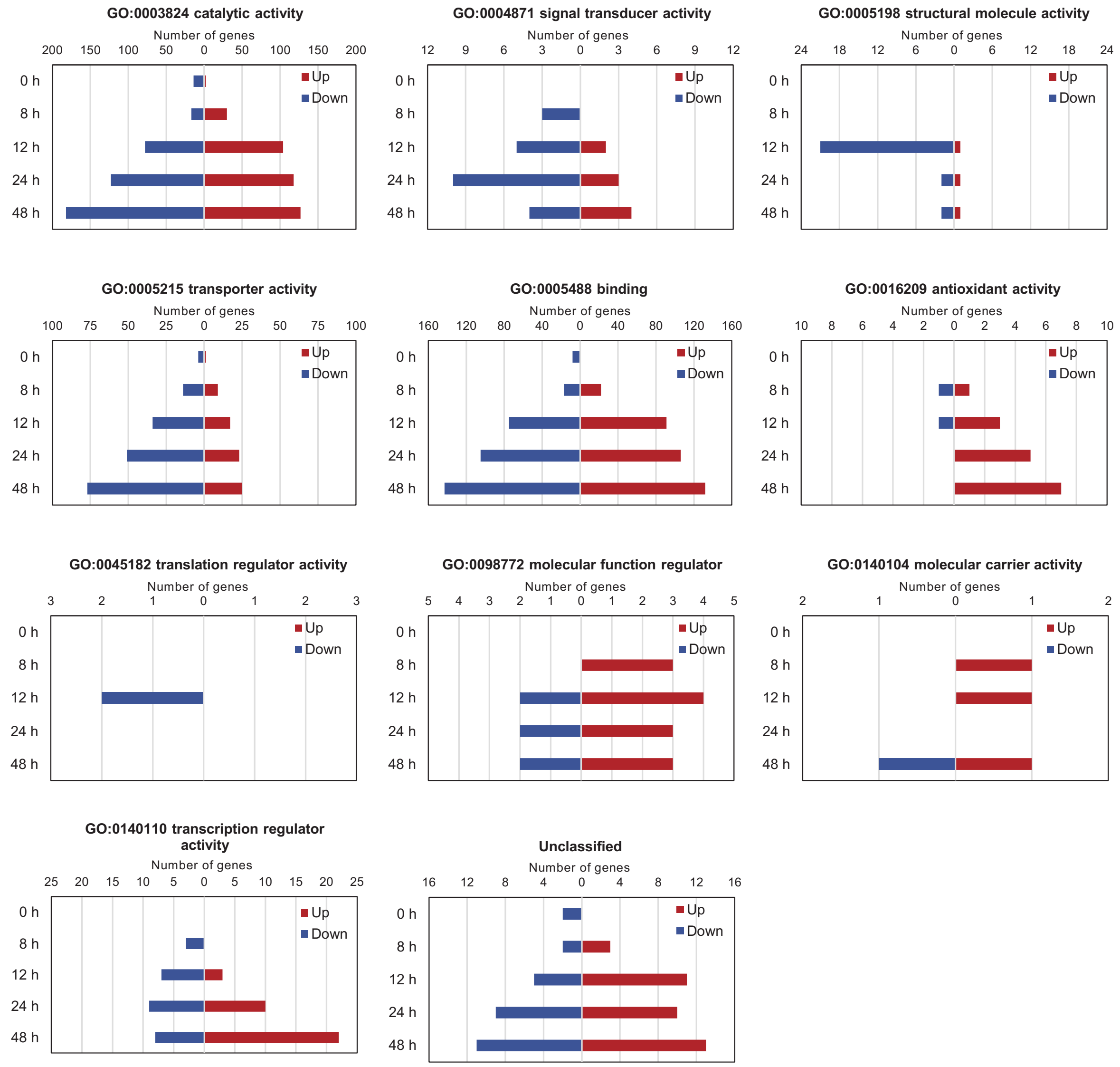

Supplementary Fig. S9 Gene Ontology categories of differentially expressed genes (DEGs) in AtDTX1-expressing cells compared to those in vector control cells at the same time points. DEGs were categorized into total (A), biological process (B), cellular process (C), and molecular function (D) based on |FC| ≥ 2 & exactTest raw *P*-value < 0.05. The number of upregulated (red bars) and downregulated (blue bars)

genes are shown separately. FC, fold-change.

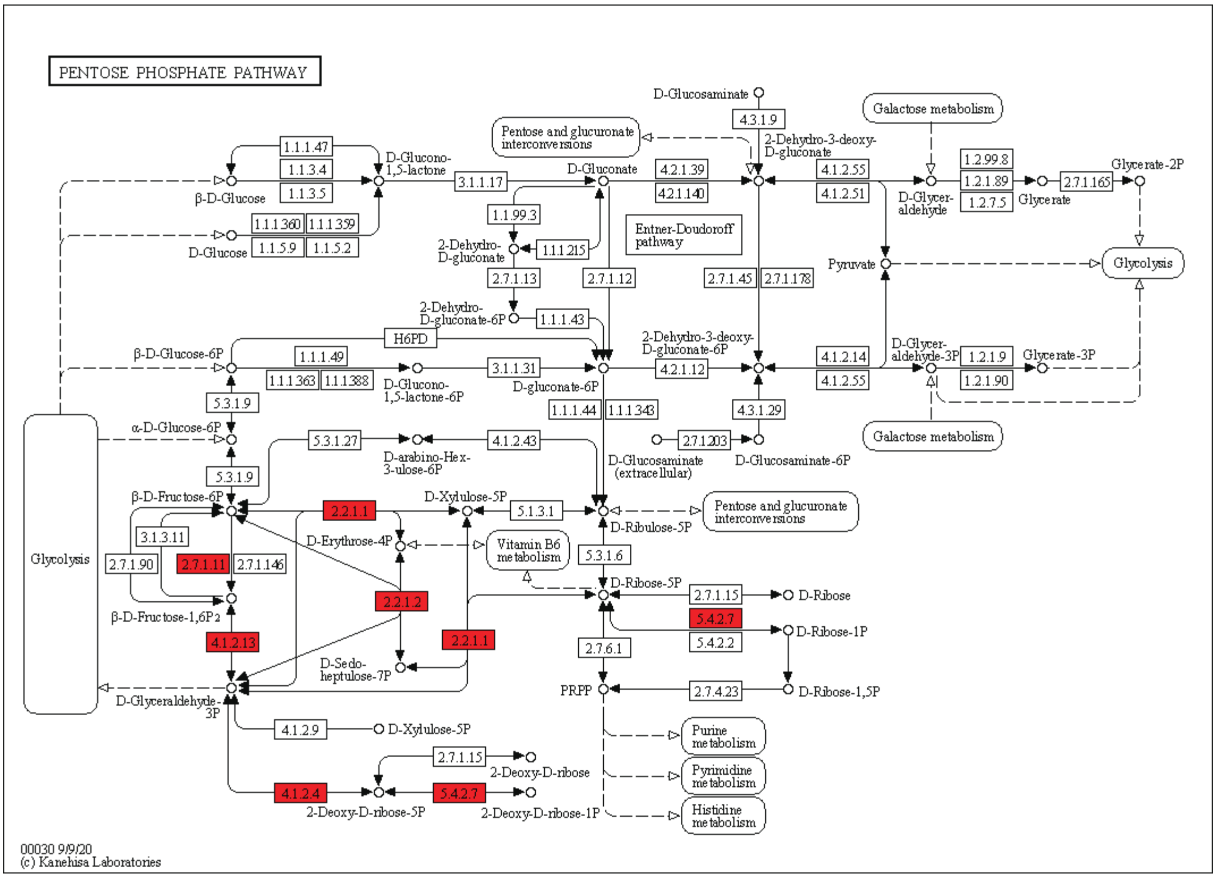
PENTOSE PHOSPHATE PATHWAY
D-Glucosaminate
4.3.1.9
Galactose metabolism
Pentose and glucuronate interconversions
2-Dehydro-3-deoxy-D-gluconate
1.1.1.47
1.1.3.4
1.1.3.5
D-Glucono-1,5-lactone
3.1.1.17
D-Gluconate
4.2.1.39
4.2.1.140
4.1.2.55
4.1.2.51
1.2.99.8
1.2.1.89
1.2.7.5
2.7.1.165
Glycerate-2P
Glycerate
D-Glyceraldehyde
β-D-Glucose
D-Glucose
1.1.1.360
1.1.1.359
1.1.5.9
1.1.5.2
1.1.99.3
2-Dehydro-D-gluconate
1.1.1.215
Entner-Doudoroff pathway
Pyruvate
Glycolysis
2.7.1.13
2.7.1.12
2.7.1.45
2.7.1.178
2-Dehydro-D-gluconate-6P
1.1.1.43
H6PD
2-Dehydro-3-deoxy-D-gluconate-6P
β-D-Glucose-6P
1.1.1.49
1.1.1.363
1.1.1.388
D-Glucono-1,5-lactone-6P
3.1.1.31
D-gluconate-6P
4.2.1.12
4.1.2.14
4.1.2.55
D-Glyceraldehyde-3P
1.2.1.9
1.2.1.90
Glycerate-3P
5.3.1.9
1.1.1.44
1.1.1.343
4.3.1.29
α-D-Glucose-6P
5.3.1.27
4.1.2.43
2.7.1.203
D-arabino-Hex-3-ulose-6P
D-Glucosaminate (extracellular)
D-Glucosamine-6P
Galactose metabolism
5.3.1.9
β-D-Fructose-6P
2.2.1.1
D-Xylulose-5P
5.1.3.1
D-Ribulose-5P
Pentose and glucuronate interconversions
3.1.3.11
D-Erythrose-4P
Vitamin B6 metabolism
Glycolysis
2.7.1.90
2.7.1.11
2.7.1.146
5.3.1.6
D-Ribose-5P
2.7.1.15
D-Ribose
2.2.1.2
5.4.2.7
β-D-Fructose-1,6P2
4.1.2.13
2.2.1.1
D-Ribose-1P
5.4.2.2
2.7.6.1
D-Sedo-heptulose-7P
PRPP
2.7.4.23
D-Ribose-1,5P
D-Glyceraldehyde-3P
4.1.2.9
D-Xylulose-5P
Purine metabolism
2.7.1.15
2-Deoxy-D-ribose
Pyrimidine metabolism
4.1.2.4
5.4.2.7
2-Deoxy-D-ribose-5P
2-Deoxy-D-ribose-1P
Histidine metabolism
00030 9/9/20
(c) Kanehisa Laboratories

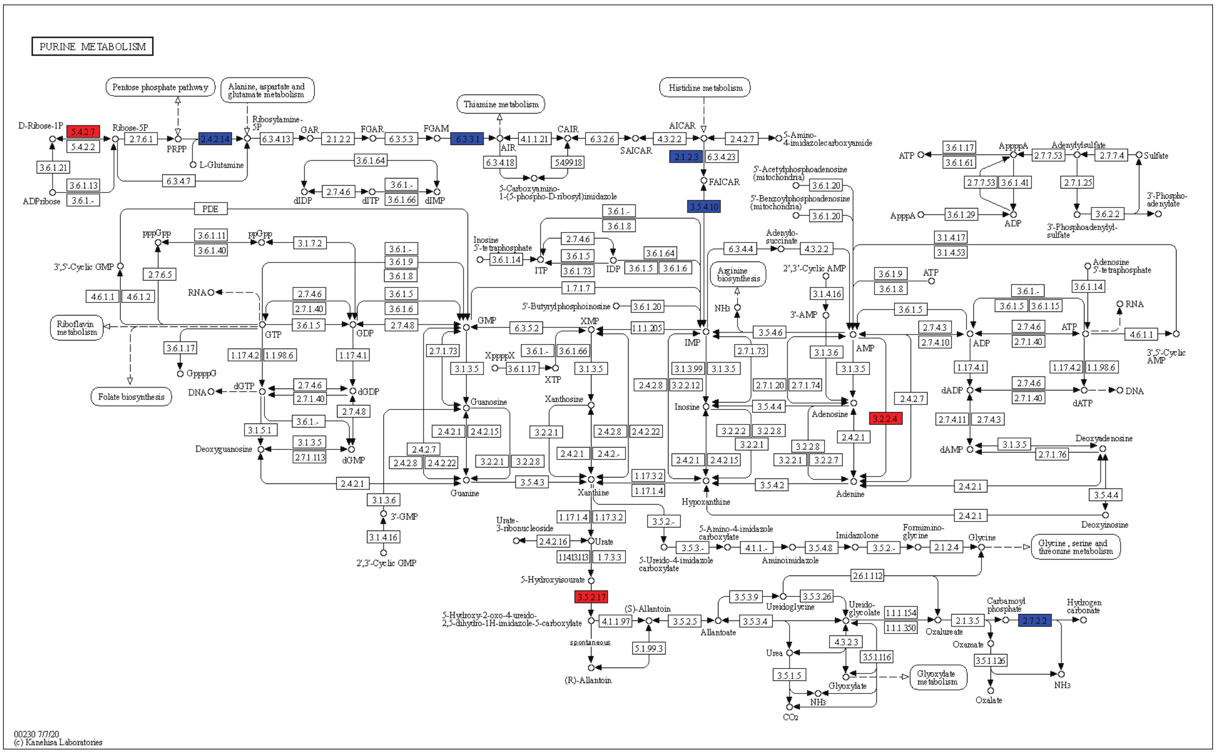
PURINE METABOLISM
Pentose phosphate pathway
Alanine, aspartate and glutamate metabolism
Thiamine metabolism
Histidine metabolism
D-Ribose-1P
Ribose-5P
PRPP
L-Glutamine
ADPribose
GAR
FGAR
FGAM
AIR
CAIR
SAICAR
AICAR
FAICAR
IMP
GMP
XMP
AMP
ADP
ATP
GTP
GDP
dGTP
dGDP
dGMP
dADP
dAMP
dATP
ITP
IDP
dIDP
dITP
dIMP
Guanosine
Xanthosine
Inosine
Adenosine
Guanine
Xanthine
Hypoxanthine
Adenine
Deoxyguanosine
Deoxyadenosine
Deoxyinosine
Urate
5-Hydroxyisourate
(S)-Allantoin
(R)-Allantoin
Allantoate
Ureidoglycine
Ureidoglycolate
Urea
Glyoxylate
Oxalureate
Oxamate
Oxalate
Carbamoyl phosphate
Hydrogen carbonate
NH3
CO2
Glycine
Glycine, serine and threonine metabolism
Glyoxylate metabolism
Riboflavin metabolism
Folate biosynthesis
Arginine biosynthesis
3',5'-Cyclic GMP
3',5'-Cyclic AMP
2',3'-Cyclic AMP
2',3'-Cyclic GMP
3'-AMP
3'-GMP
pppGpp
ppGpp
GppppG
RNA
DNA
ApppA
Adenylylsulfate
Sulfate
PDE
00230 7/7/20
(c) Kanehisa Laboratories

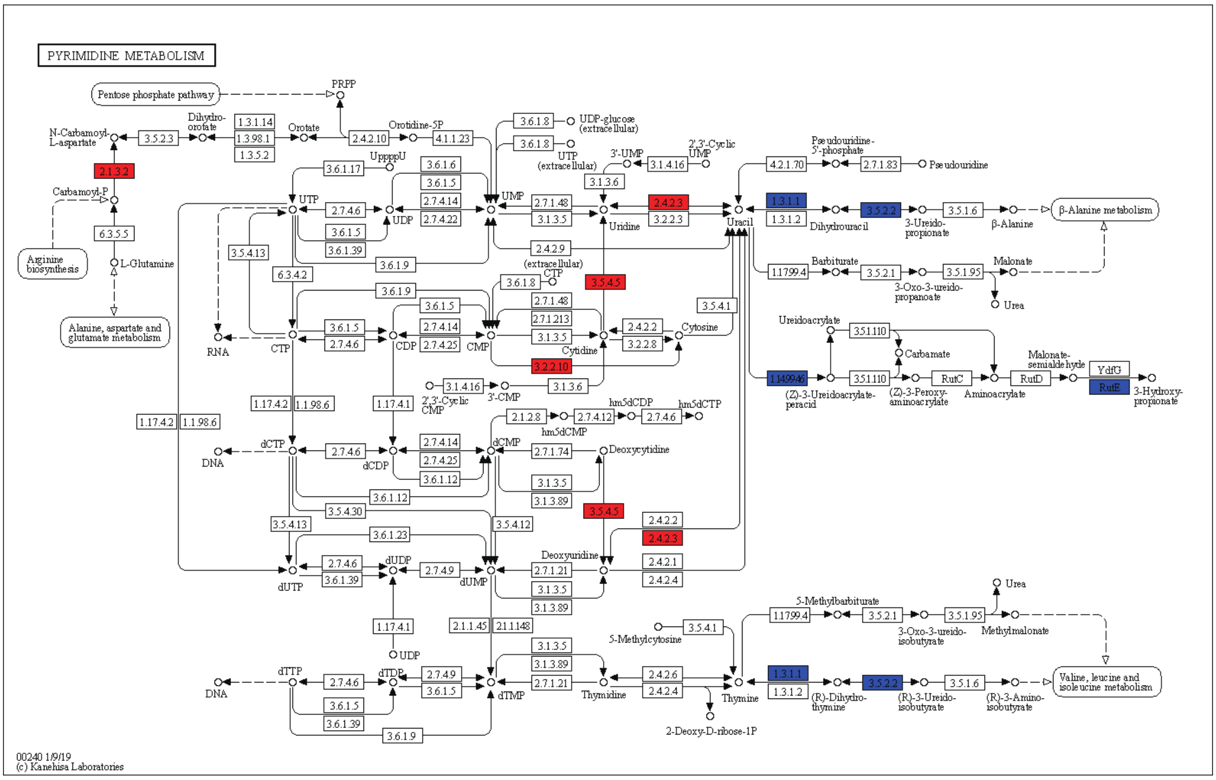
PYRIMIDINE METABOLISM
Pentose phosphate pathway
PRPP
N-Carbamoyl-L-aspartate
Dihydro-orotate
Orotate
Orotidine-5P
UpppppU
Carbamoyl-P
L-Glutamine
Arginine biosynthesis
Alanine, aspartate and glutamate metabolism
UTP
UDP
UMP
CTP
CDP
CMP
RNA
UDP-glucose (extracellular)
UTP (extracellular)
CTP (extracellular)
3'-UMP
2',3'-Cyclic UMP
Uridine
Uracil
Cytidine
Cytosine
2',3'-Cyclic CMP
3'-CMP
hm5dCDP
hm5dCTP
hm5dCMP
dCTP
dCDP
dCMP
Deoxycytidine
DNA
dUTP
dUDP
dUMP
Deoxyuridine
UDP
dTTP
dTDP
dTMP
Thymidine
Thymine
5-Methylcytosine
2-Deoxy-D-ribose-1P
Pseudouridine-5'-phosphate
Pseudouridine
Dihydrouracil
3-Ureido-propionate
β-Alanine
β-Alanine metabolism
Barbiturate
3-Oxo-3-ureido-propanoate
Malonate
Urea
Ureidoacrylate
Carbamate
Malonate-semialdehyde
(Z)-3-Ureidoacrylate-peracid
(Z)-3-Peroxy-aminoacrylate
Aminoacrylate
3-Hydroxy-propionate
YdfG
RutE
RutC
RutD
5-Methylbarbiturate
3-Oxo-3-ureido-isobutyrate
Methylmalonate
Valine, leucine and isoleucine metabolism
(R)-Dihydro-thymine
(R)-3-Ureido-isobutyrate
(R)-3-Amino-isobutyrate
2.1.3.2
3.5.2.3
1.3.1.14
1.3.98.1
1.3.5.2
2.4.2.10
4.1.1.23
6.3.5.5
3.6.1.17
3.6.1.6
3.6.1.5
2.7.4.14
2.7.4.22
2.7.4.6
3.6.1.39
3.6.1.9
3.5.4.13
6.3.4.2
3.6.1.8
3.1.4.16
3.1.3.6
2.7.1.48
3.1.3.5
2.4.2.3
3.2.2.3
2.4.2.9
3.5.4.5
2.7.1.213
2.7.4.25
2.4.2.2
3.2.2.8
3.5.4.1
3.2.2.10
1.17.4.2
1.1.98.6
1.17.4.1
2.1.2.8
2.7.4.12
2.7.1.74
3.1.3.89
3.6.1.12
3.5.4.30
3.6.1.23
3.5.4.12
2.7.4.9
2.7.1.21
2.4.2.1
2.4.2.4
2.1.1.45
2.1.1.148
2.4.2.6
4.2.1.70
2.7.1.83
1.3.1.1
1.3.1.2
3.5.2.2
3.5.1.6
1.17.99.4
3.5.2.1
3.5.1.95
3.5.1.110
1.14.99.46
00240 1/9/19
(c) Kanehisa Laboratories

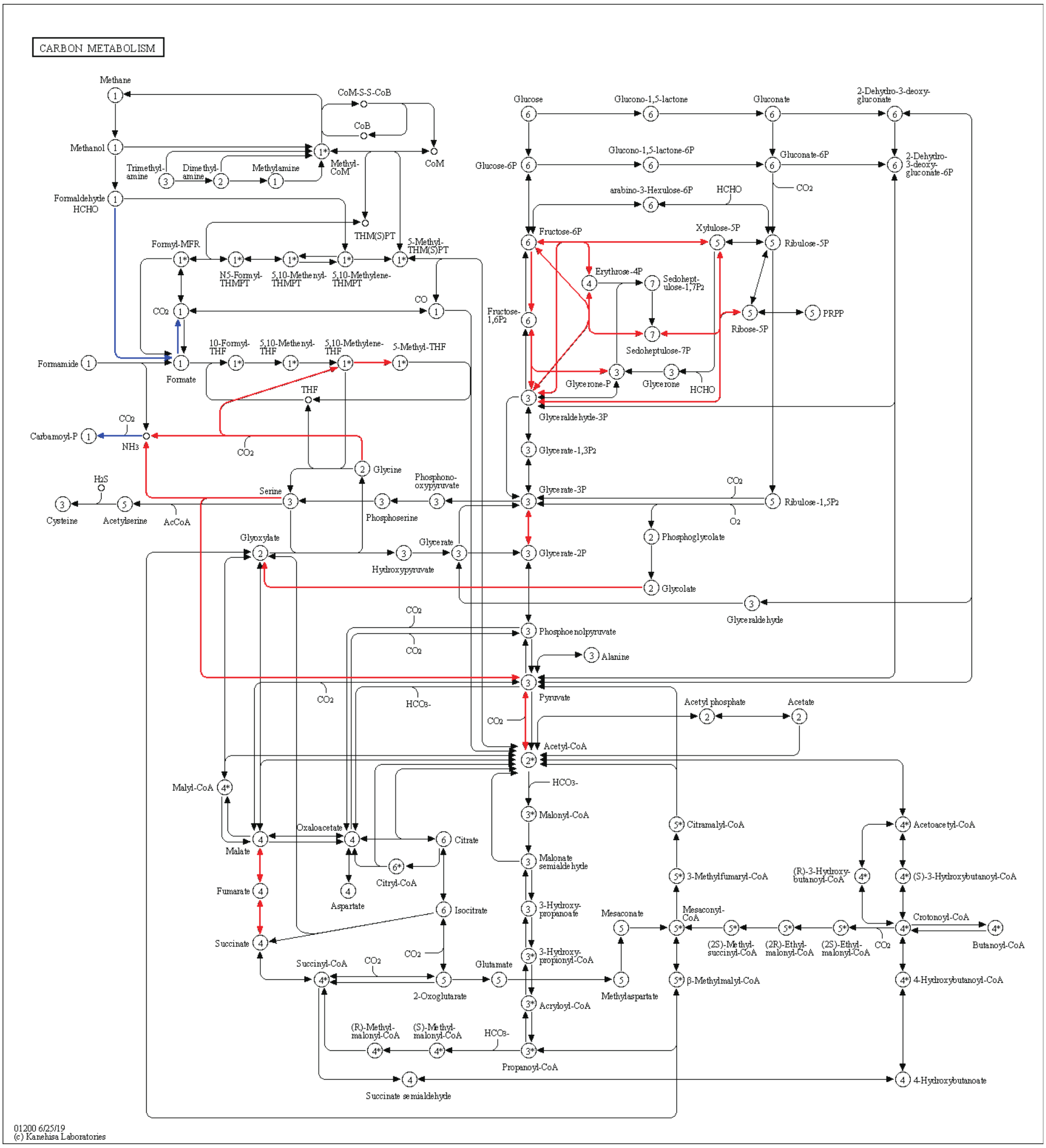

CARBON METABOLISM
Methane
CoM-S-S-CoB
CoB
CoM
Methanol
Trimethyl-amine
Dimethyl-amine
Methylamine
Methyl-CoM
Formaldehyde HCHO
THM(S)PT
5-Methyl-THM(S)PT
Formyl-MFR
N5-Formyl-THMPT
5,10-Methenyl-THMPT
5,10-Methylene-THMPT
CO
CO_2
Formamide
Formate
10-Formyl-THF
5,10-Methenyl-THF
5,10-Methylene-THF
5-Methyl-THF
THF
Carbamoyl-P
NH3
Glycine
Serine
Phosphoserine
Phospho-oxypyruvate
H2S
Cysteine
Acetylserine
AcCoA
Glyoxylate
Hydroxypyruvate
Glycerate
Glucose
Glucono-1,5-lactone
Gluconate
2-Dehydro-3-deoxy-gluconate
Glucose-6P
Glucono-1,5-lactone-6P
Gluconate-6P
2-Dehydro-3-deoxy-gluconate-6P
arabino-3-Hexulose-6P
HCHO
Fructose-6P
Xylulose-5P
Ribulose-5P
Erythrose-4P
Sedohept-ulose-1,7P2
Fructose-1,6P2
Ribose-5P
PRPP
Sedoheptulose-7P
Glycerone-P
Glycerone
Glyceraldehyde-3P
Glycerate-1,3P2
Glycerate-3P
Ribulose-1,5P2
O2
Phosphoglycolate
Glycerate-2P
Glycolate
Glyceraldehyde
Phosphoenolpyruvate
Alanine
Pyruvate
HCO3-
Acetyl phosphate
Acetate
Acetyl-CoA
Malyl-CoA
Malonyl-CoA
Malonate semialdehyde
Oxaloacetate
Citrate
Malate
Citryl-CoA
Aspartate
Fumarate
Isocitrate
Succinate
3-Hydroxy-propanoate
3-Hydroxy-propionyl-CoA
Succinyl-CoA
2-Oxoglutarate
Glutamate
Mesaconate
Methylaspartate
Acryloyl-CoA
(R)-Methyl-malonyl-CoA
(S)-Methyl-malonyl-CoA
Propanoyl-CoA
Succinate semialdehyde
Citramalyl-CoA
3-Methylfumaryl-CoA
Mesaconyl-CoA
β-Methylmalyl-CoA
(2S)-Methyl-succinyl-CoA
(2R)-Ethyl-malonyl-CoA
(2S)-Ethyl-malonyl-CoA
Acetoacetyl-CoA
(R)-3-Hydroxy-butanoyl-CoA
(S)-3-Hydroxybutanoyl-CoA
Crotonoyl-CoA
Butanoyl-CoA
4-Hydroxybutanoyl-CoA
4-Hydroxybutanoate
01200 6/25/19
(c) Kanehisa Laboratories

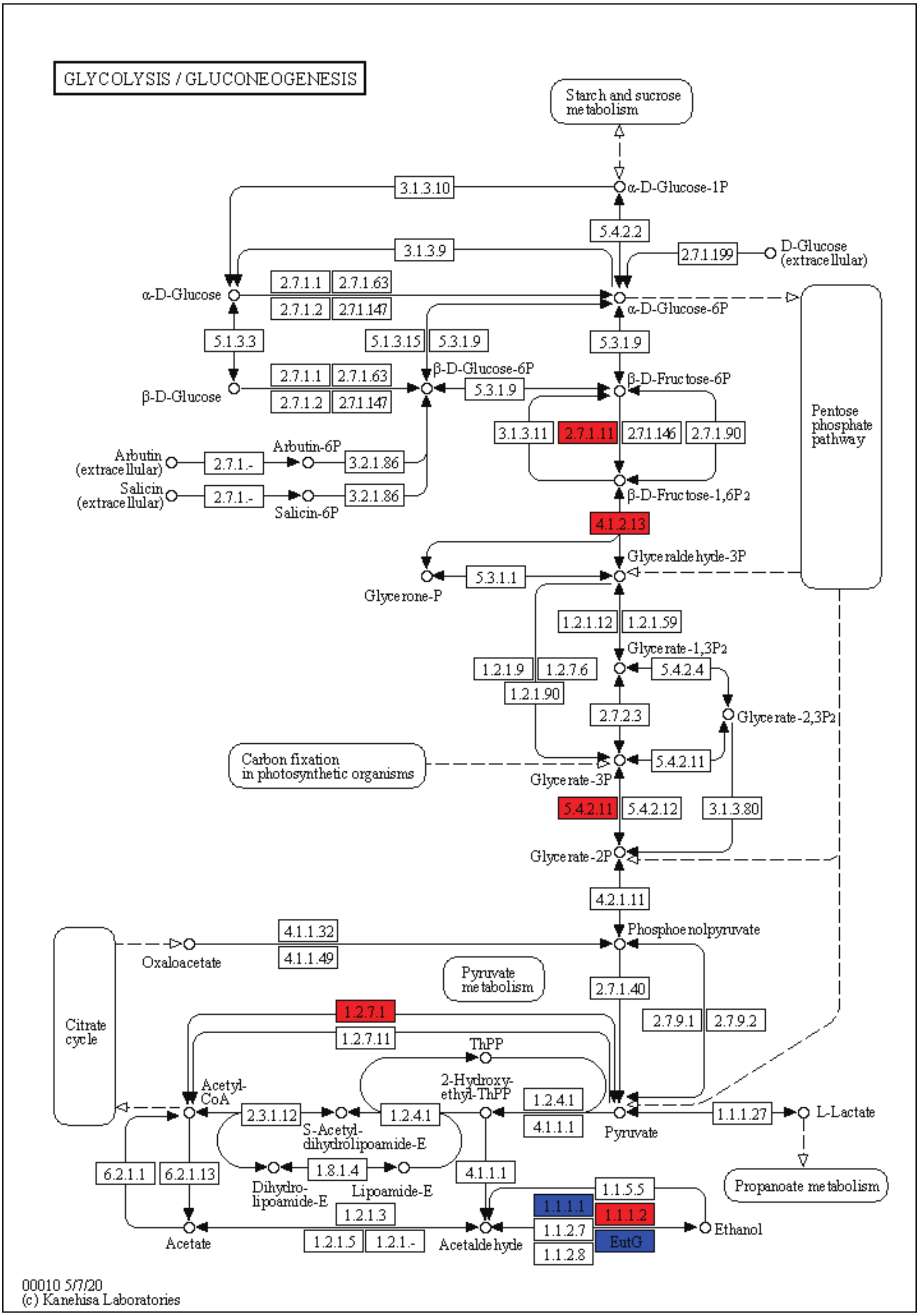
GLYCOLYSIS / GLUCONEOGENESIS
Starch and sucrose metabolism
α-D-Glucose-1P
3.1.3.10
5.4.2.2
3.1.3.9
2.7.1.199
D-Glucose (extracellular)
α-D-Glucose
2.7.1.1
2.7.1.63
2.7.1.2
2.7.1.147
α-D-Glucose-6P
5.1.3.3
5.1.3.15
5.3.1.9
β-D-Glucose
β-D-Glucose-6P
β-D-Fructose-6P
Pentose phosphate pathway
3.1.3.11
2.7.1.11
2.7.1.146
2.7.1.90
Arbutin (extracellular)
2.7.1.-
Arbutin-6P
3.2.1.86
Salicin (extracellular)
Salicin-6P
β-D-Fructose-1,6P2
4.1.2.13
Glyceraldehyde-3P
Glycerone-P
5.3.1.1
1.2.1.12
1.2.1.59
Glycerate-1,3P2
1.2.1.9
1.2.7.6
5.4.2.4
1.2.1.90
2.7.2.3
Glycerate-2,3P2
Carbon fixation in photosynthetic organisms
Glycerate-3P
5.4.2.11
5.4.2.12
3.1.3.80
Glycerate-2P
4.2.1.11
Phosphoenolpyruvate
Citrate cycle
Oxaloacetate
4.1.1.32
4.1.1.49
Pyruvate metabolism
2.7.1.40
1.2.7.1
1.2.7.11
2.7.9.1
2.7.9.2
ThPP
Acetyl-CoA
2-Hydroxy-ethyl-ThPP
1.2.4.1
2.3.1.12
S-Acetyl-dihydrolipoamide-E
4.1.1.1
Pyruvate
1.1.1.27
L-Lactate
6.2.1.1
6.2.1.13
1.8.1.4
Dihydro-lipoamide-E
Lipoamide-E
Propanoate metabolism
1.1.5.5
1.1.1.1
1.1.1.2
1.2.1.3
1.1.2.7
EutG
1.1.2.8
Ethanol
Acetate
1.2.1.5
1.2.1.-
Acetaldehyde
00010 5/7/20
(c) Kanehisa Laboratories

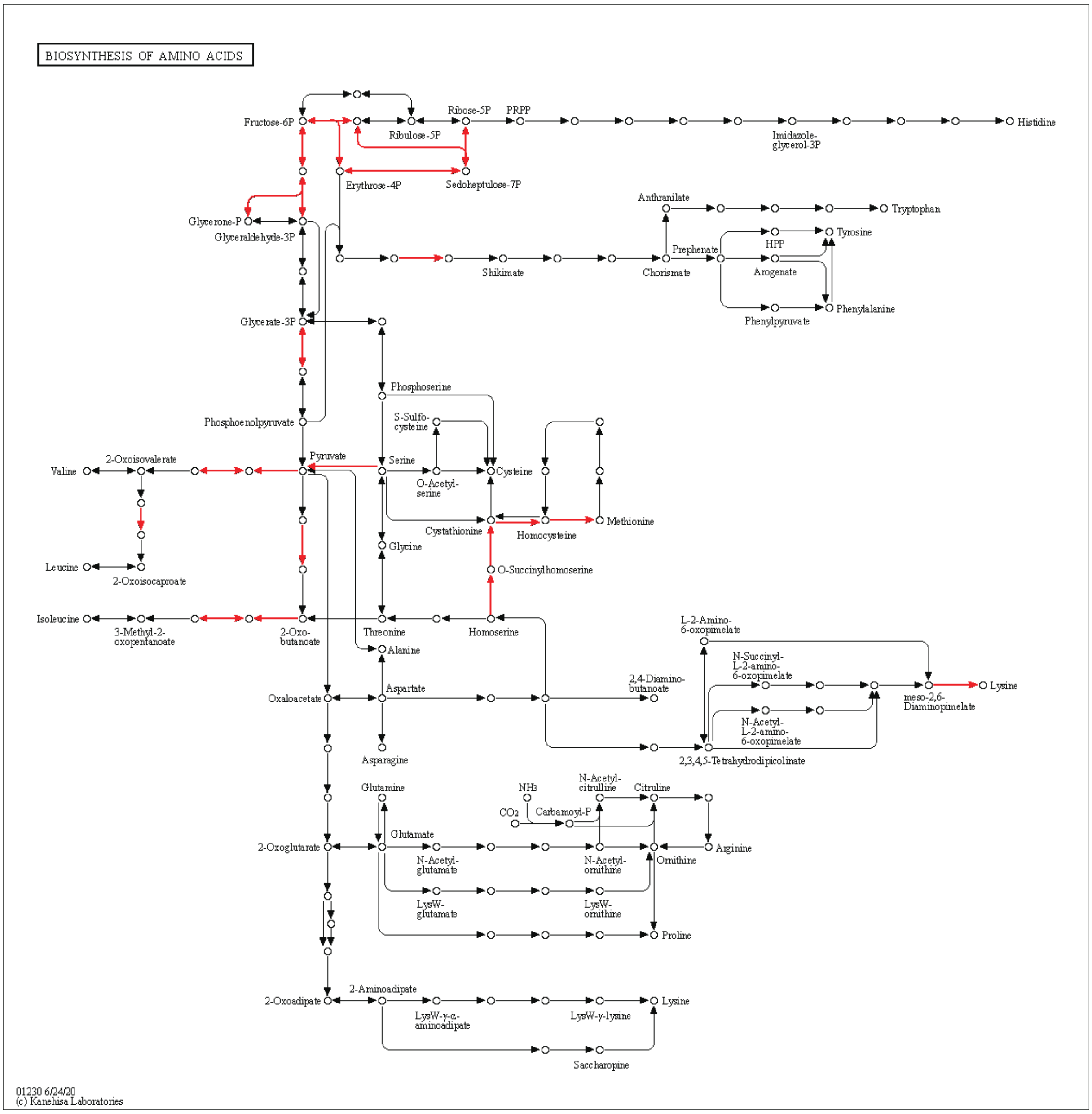
BIOSYNTHESIS OF AMINO ACIDS
Fructose-6P
Ribose-5P
PRPP
Ribulose-5P
Imidazole-glycerol-3P
Histidine
Erythrose-4P
Sedoheptulose-7P
Glycerone-P
Glyceraldehyde-3P
Anthranilate
Tryptophan
Tyrosine
HPP
Prephenate
Shikimate
Chorismate
Arogenate
Phenylalanine
Phenylpyruvate
Glycerate-3P
Phosphoserine
S-Sulfo-cysteine
Phosphoenolpyruvate
2-Oxoisovalerate
Valine
Pyruvate
Serine
Cysteine
O-Acetyl-serine
Methionine
Cystathionine
Homocysteine
Glycine
Leucine
2-Oxoisocaproate
O-Succinylhomoserine
Isoleucine
3-Methyl-2-oxopentanoate
2-Oxo-butanoate
Threonine
Homoserine
L-2-Amino-6-oxopimelate
Alanine
N-Succinyl-L-2-amino-6-oxopimelate
2,4-Diamino-butanoate
Aspartate
Oxaloacetate
Lysine
meso-2,6-Diaminopimelate
N-Acetyl-L-2-amino-6-oxopimelate
Asparagine
2,3,4,5-Tetrahydrodipicolinate
Glutamine
NH3
N-Acetyl-citrulline
Citruline
CO2
Carbamoyl-P
Glutamate
2-Oxoglutarate
Arginine
N-Acetyl-glutamate
N-Acetyl-ornithine
Ornithine
LysW-glutamate
LysW-ornithine
Proline
2-Aminoadipate
2-Oxoadipate
Lysine
LysW-γ-α-aminoadipate
LysW-γ-lysine
Saccharopine
01230 6/24/20
(c) Kanehisa Laboratories

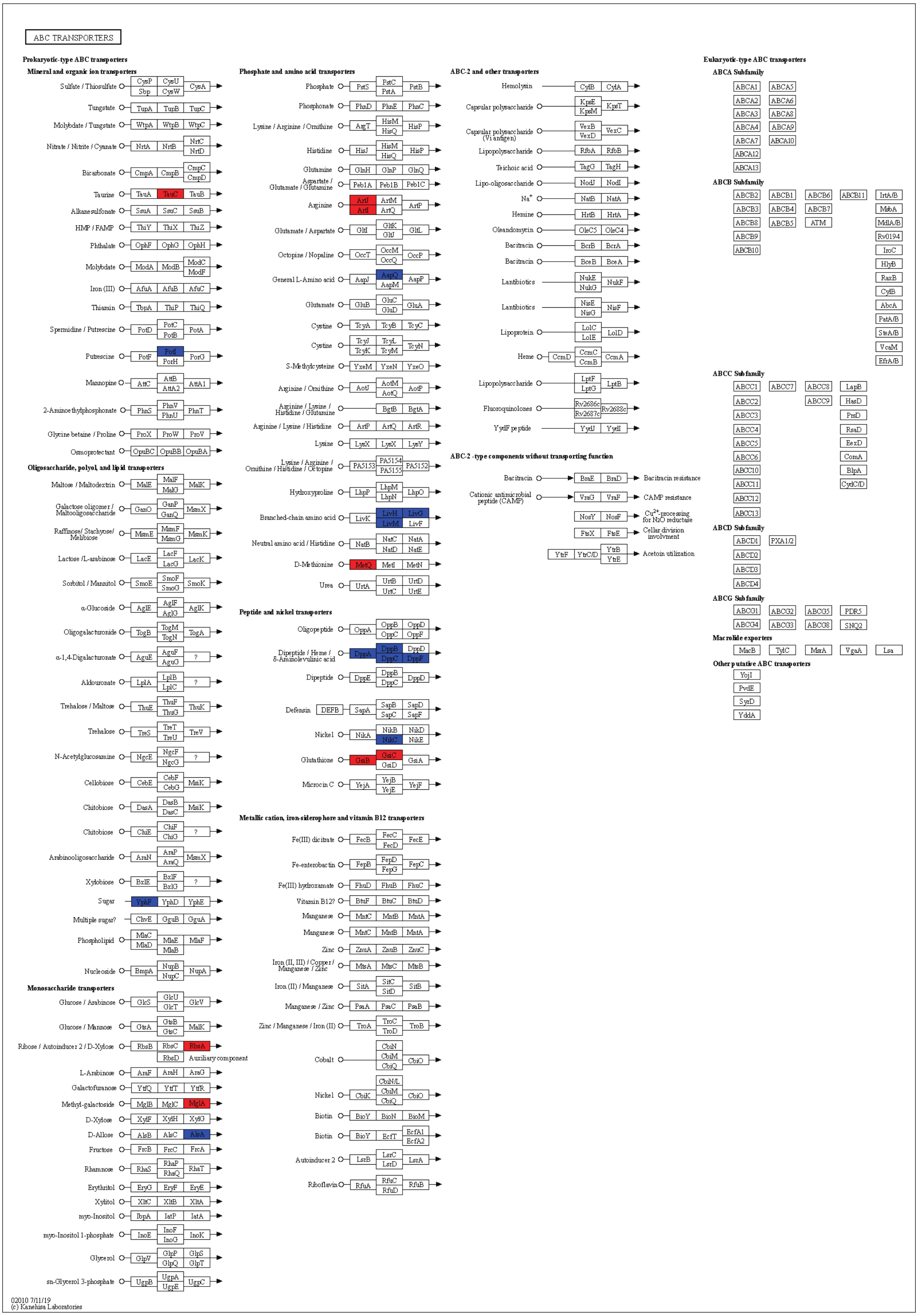

ABC TRANSPORTERS
Prokaryotic-type ABC transporters
Mineral and organic ion transporters
Sulfate / Thiosulfate CysP Sbp CysU CysW CysA
Tungstate TupA TupB TupC
Molybdate / Tungstate WtpA WtpB WtpC
Nitrate / Nitrite / Cyanate NrtA NrtB NrtC NrtD
Bicarbonate CmpA CmpB CmpC CmpD
Taurine TauA TauC TauB
Alkanesulfonate SsuA SsuC SsuB
HMP / FAMP ThiY ThiX ThiZ
Phthalate OphF OphG OphH
Molybdate ModA ModB ModC ModF
Iron (III) AfuA AfuB AfuC
Thiamin TbpA ThiP ThiQ
Spermidine / Putrescine PotD PotC PotB PotA
Putrescine PotF PotI PotH PotG
Mannopine AttC AttB AttA2 AttA1
2-Aminoethylphosphonate PhnS PhnV PhnU PhnT
Glycine betaine / Proline ProX ProW ProV
Osmoprotectant OpuBC OpuBB OpuBA
Oligosaccharide, polyol, and lipid transporters
Maltose / Maltodextrin MalE MalF MalG MalK
Galactose oligomer / Maltooligosaccharide GanO GanP GanQ MsmX
Raffinose/ Stachyose/ Melibiose MsmE MsmF MsmG MsmK
Lactose /L-arabinose LacE LacF LacG LacK
Sorbitol / Mannitol SmoE SmoF SmoG SmoK
α-Glucoside AglE AglF AglG AglK
Oligogalacturonide TogB TogM TogN TogA
α-1,4-Digalacturonate AguE AguF AguG ?
Aldouronate LplA LplB LplC ?
Trehalose / Maltose ThuE ThuF ThuG ThuK
Trehalose TreS TreT TreU TreV
N-Acetylglucosamine NgcE NgcF NgcG ?
Cellobiose CebE CebF CebG MsiK
Chitobiose DasA DasB DasC MsiK
Chitobiose ChiE ChiF ChiG ?
Arabinooligosaccharide AraN AraP AraQ MsmX
Xylobiose BxlE BxlF BxlG ?
Sugar YphF YphD YphE
Multiple sugar? ChvE GguB GguA
Phospholipid MlaC MlaD MlaE MlaF MlaB
Nucleoside BmpA NupB NupC NupA
Monosaccharide transporters
Glucose / Arabinose GlcS GlcU GlcT GlcV
Glucose / Mannose GtsA GtsB GtsC MalK
Ribose / Autoinducer 2 / D-Xylose RbsB RbsC RbsA RbsD Auxiliary component
L-Arabinose AraF AraH AraG
Galactofuranose YtfQ YtfT YtfR
Methyl-galactoside MglB MglC MglA
D-Xylose XylF XylH XylG
D-Allose AlsB AlsC AlsA
Fructose FrcB FrcC FrcA
Rhamnose RhaS RhaP RhaQ RhaT
Erythritol EryG EryF EryE
Xylitol XltC XltB XltA
myo-Inositol IbpA IatP IatA
myo-Inositol 1-phosphate InoE InoF InoG InoK
Glycerol GlpV GlpP GlpQ GlpS GlpT
sn-Glycerol 3-phosphate UgpB UgpA UgpE UgpC
Phosphate and amino acid transporters
Phosphate PstS PstC PstA PstB
Phosphonate PhnD PhnE PhnC
Lysine / Arginine / Ornithine ArgT HisM HisQ HisP
Histidine HisJ HisM HisQ HisP
Glutamine GlnH GlnP GlnQ
Aspartate / Glutamate / Glutamine Peb1A Peb1B Peb1C
Arginine ArtI ArtJ ArtM ArtQ ArtP
Glutamate / Aspartate GltI GltK GltJ GltL
Octopine / Nopaline OccT OccM OccQ OccP
General L-Amino acid AapJ AapQ AapM AapP
Glutamate GluB GluC GluD GluA
Cystine TcyA TcyB TcyC
Cystine TcyJ TcyK TcyL TcyM TcyN
S-Methylcysteine YxeM YxeN YxeO
Arginine / Ornithine AotJ AotM AotQ AotP
Arginine / Lysine / Histidine / Glutamine BgtB BgtA
Arginine / Lysine / Histidine ArtP ArtQ ArtR
Lysine LysX LysX LysY
Lysine / Arginine / Ornithine / Histidine / Octopine PA5153 PA5154 PA5155 PA5152
Hydroxyproline LhpP LhpM LhpN LhpO
Branched-chain amino acid LivK LivH LivM LivG LivF
Neutral amino acid / Histidine NatB NatC NatD NatA NatE
D-Methionine MetQ MetI MetN
Urea UrtA UrtB UrtC UrtD UrtE
Peptide and nickel transporters
Oligopeptide OppA OppB OppC OppD OppF
Dipeptide / Heme / δ-Aminolevulinic acid DppA DppB DppC DppD DppF
Dipeptide DppE DppB DppC DppD
Defensin DEFB SapA SapB SapC SapD SapF
Nickel NikA NikB NikC NikD NikE
Glutathione GsiB GsiC GsiD GsiA
Microcin C YejA YejB YejE YejF
Metallic cation, iron-siderophore and vitamin B12 transporters
Fe(III) dicitrate FecB FecC FecD FecE
Fe-enterobactin FepB FepD FepG FepC
Fe(III) hydroxamate FhuD FhuB FhuC
Vitamin B12? BtuF BtuC BtuD
Manganese MntC MntB MntA
Manganese MntC MntB MntA
Zinc ZnuA ZnuB ZnuC
Iron (II, III) / Copper / Manganese / Zinc MtsA MtsC MtsB
Iron (II) / Manganese SitA SitC SitD SitB
Manganese / Zinc PsaA PsaC PsaB
Zinc / Manganese / Iron (II) TroA TroC TroD TroB
Cobalt CbiN CbiM CbiQ CbiO
Nickel CbiK CbiN/L CbiM CbiQ CbiO
Biotin BioY BioN BioM
Biotin BioY EcfT EcfA1 EcfA2
Autoinducer 2 LsrB LsrC LsrD LsrA
Riboflavin RfuA RfuC RfuD RfuB
ABC-2 and other transporters
Hemolysin CylB CylA
Capsular polysaccharide KpsE KpsM KpsT
Capsular polysaccharide (Vi antigen) VexB VexD VexC
Lipopolysaccharide RfbA RfbB
Teichoic acid TagG TagH
Lipo-oligosaccharide NodJ NodI
Na+ NatB NatA
Hemine HrtB HrtA
Oleandomycin OleC5 OleC4
Bacitracin BcrB BcrA
Bacitracin BceB BceA
Lantibiotics NukE NukG NukF
Lantibiotics NisE NisG NisF
Lipoprotein LolC LolE LolD
Heme CcmD CcmC CcmB CcmA
Lipopolysaccharide LptF LptG LptB
Fluoroquinolones Rv2686c Rv2687c Rv2688c
YydF peptide YydJ YydI
ABC-2 -type components without transporting function
Bacitracin BraE BraD Bacitracin resistance
Cationic antimicrobial peptide (CAMP) VraG VraF CAMP resistance
NosY NosF Cu2+-processing for N2O reductase
FtsX FtsE Cellar division involvment
YtrF YtrC/D YtrB YtrE Acetoin utilization
Eukaryotic-type ABC transporters
ABCA Subfamily
ABCA1 ABCA5 ABCA2 ABCA6 ABCA3 ABCA8 ABCA4 ABCA9 ABCA7 ABCA10 ABCA12 ABCA13
ABCB Subfamily
ABCB2 ABCB1 ABCB6 ABCB11 ABCB3 ABCB4 ABCB7 ABCB8 ABCB5 ATM ABCB9 ABCB10
IrtA/B MsbA MdlA/B Rv0194 IrtC HlyB RaxB CydB AbcA PstA/B SteA/B VcaM EfrA/B
ABCC Subfamily
ABCC1 ABCC7 ABCC8 LapB ABCC2 ABCC9 HasD ABCC3 PrtD ABCC4 RsaD ABCC5 EexD ABCC6 ComA ABCC10 BlpA ABCC11 CydC/D ABCC12 ABCC13
ABCD Subfamily
ABCD1 PXA1/2 ABCD2 ABCD3 ABCD4
ABCG Subfamily
ABCG1 ABCG2 ABCG5 PDR5 ABCG4 ABCG3 ABCG8 SNQ2
Macrolide exporters
MacB TylC MsrA VgaA Lsa
Other putative ABC transporters
YojI PvdE SyrD YddA
02010 7/11/19
(c) Kanehisa Laboratories

Supplementary Fig. S10 Representative metabolic pathway maps at 12 h from KEGG mapper. Red indicates upregulation and blue indicates downregulation of each gene.

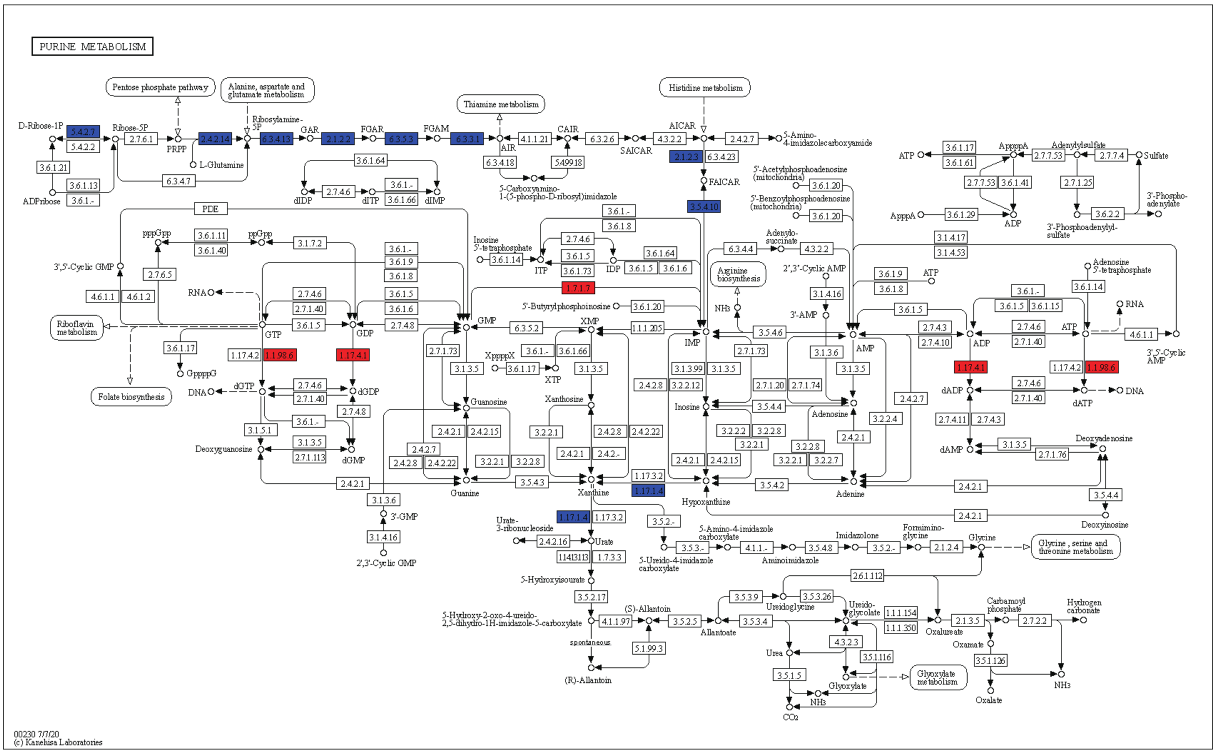
PURINE METABOLISM
Pentose phosphate pathway
Alanine, aspartate and glutamate metabolism
Thiamine metabolism
Histidine metabolism
D-Ribose-1P
Ribose-5P
PRPP
L-Glutamine
Ribosylamine-5P
GAR
FGAR
FGAM
AIR
CAIR
SAICAR
AICAR
5-Amino-4-imidazolecarboxyamide
5-Carboxyamino-1-(5-phospho-D-ribosyl)imidazole
FAICAR
ADPribose
dIDP
dITP
dIMP
PDE
pppGpp
ppGpp
3',5'-Cyclic GMP
RNA
Riboflavin metabolism
Folate biosynthesis
GTP
GDP
GMP
GppppG
dGTP
dGDP
dGMP
DNA
Deoxyguanosine
Guanosine
Guanine
Inosine 5'-triphosphate
ITP
IDP
IMP
XppppX
XTP
XMP
Xanthosine
Xanthine
5'-Butyrylphosphoinosine
Inosine
Hypoxanthine
3'-GMP
2',3'-Cyclic GMP
Urate-3-ribonucleoside
Urate
5-Hydroxyisourate
5-Hydroxy-2-oxo-4-ureido-2,5-dihydro-1H-imidazole-5-carboxylate
spontaneous
(R)-Allantoin
(S)-Allantoin
Allantoate
5-Amino-4-imidazole carboxylate
5-Ureido-4-imidazole carboxylate
Aminoimidazole
Imidazolone
Formimino-glycine
Glycine
Glycine, serine and threonine metabolism
Ureidoglycine
Ureido-glycolate
Oxalureate
Carbamoyl phosphate
Hydrogen carbonate
Oxamate
Oxalate
Urea
Glyoxylate
Glyoxylate metabolism
NH3
CO2
5'-Acetylphosphoadenosine (mitochondria)
5'-Benzoylphosphoadenosine (mitochondria)
Adenylo-succinate
Arginine biosynthesis
2',3'-Cyclic AMP
3'-AMP
AMP
Adenosine
Adenine
ADP
ATP
dADP
dATP
dAMP
Deoxyadenosine
Deoxyinosine
Adenosine 5'-tetraphosphate
3',5'-Cyclic AMP
ApppA
Adenylylsulfate
Sulfate
3'-Phospho-adenylate
3'-Phosphoadenylyl-sulfate
ApppA
00230 7/7/20
(c) Kanehisa Laboratories

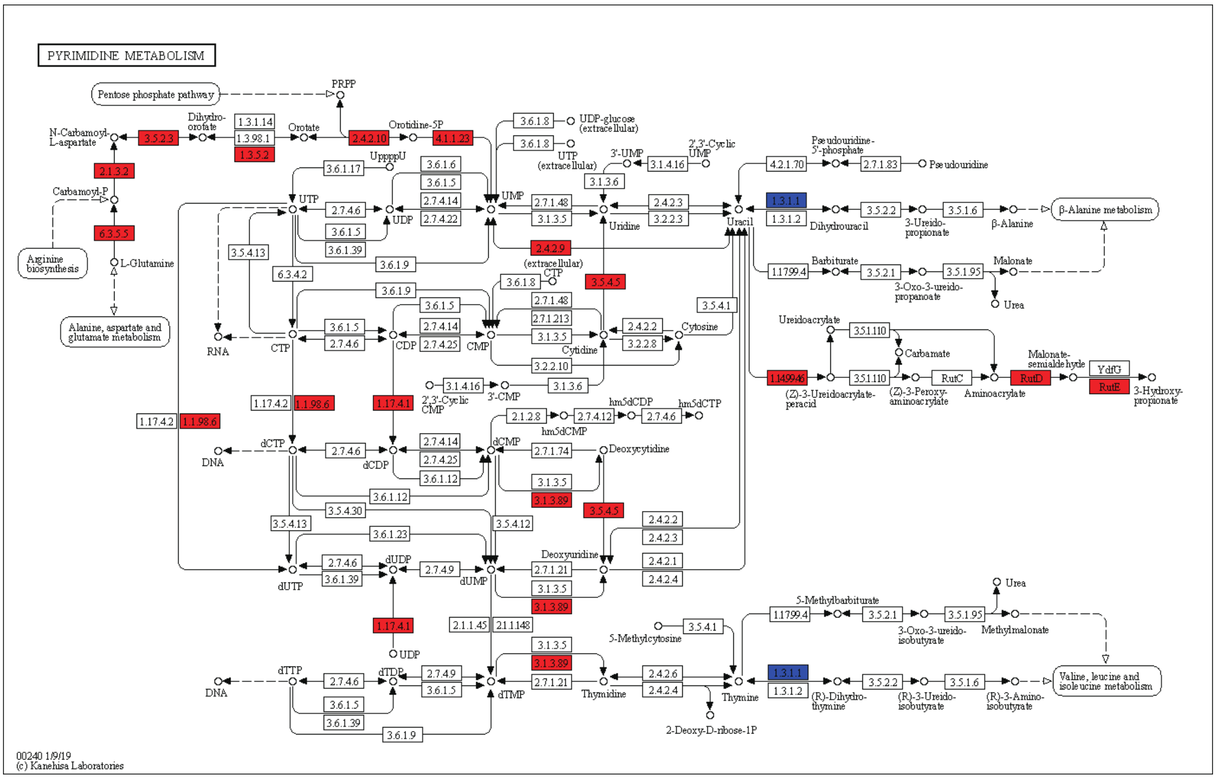
PYRIMIDINE METABOLISM
Pentose phosphate pathway
PRPP
N-Carbamoyl-L-aspartate
Dihydro-orotate
Orotate
Orotidine-5P
3.5.2.3
1.3.1.14
1.3.98.1
1.3.5.2
2.4.2.10
4.1.1.23
2.1.3.2
Carbamoyl-P
6.3.5.5
L-Glutamine
Arginine biosynthesis
Alanine, aspartate and glutamate metabolism
UpppU
3.6.1.17
UTP
UDP
UMP
2.7.4.6
3.6.1.6
3.6.1.5
2.7.4.14
2.7.4.22
3.6.1.5
3.6.1.39
3.5.4.13
6.3.4.2
3.6.1.9
3.6.1.8
UDP-glucose (extracellular)
UTP (extracellular)
3'-UMP
2',3'-Cyclic UMP
3.1.4.16
3.1.3.6
2.7.1.48
3.1.3.5
Uridine
2.4.2.3
3.2.2.3
Uracil
2.4.2.9
(extracellular) CTP
3.5.4.5
3.6.1.8
2.7.1.48
2.7.1.213
3.1.3.5
3.2.2.10
Cytidine
2.4.2.2
3.2.2.8
Cytosine
3.5.4.1
RNA
CTP
CDP
CMP
3.6.1.5
2.7.4.6
3.6.1.5
2.7.4.14
2.7.4.25
2',3'-Cyclic CMP
3.1.4.16
3'-CMP
3.1.3.6
1.17.4.2
1.1.98.6
1.17.4.1
DNA
dCTP
dCDP
dCMP
2.7.4.6
2.7.4.14
2.7.4.25
3.6.1.12
2.1.2.8
hm5dCMP
2.7.4.12
hm5dCDP
2.7.4.6
hm5dCTP
2.7.1.74
Deoxycytidine
3.1.3.5
3.1.3.89
3.5.4.5
3.5.4.30
3.5.4.13
3.6.1.23
3.5.4.12
2.4.2.2
2.4.2.3
dUTP
dUDP
dUMP
2.7.4.6
3.6.1.39
2.7.4.9
2.7.1.21
Deoxyuridine
3.1.3.5
3.1.3.89
2.4.2.1
2.4.2.4
1.17.4.1
UDP
2.1.1.45
2.1.1.148
5-Methylcytosine
3.5.4.1
DNA
dTTP
dTDP
dTMP
2.7.4.6
2.7.4.9
3.6.1.5
3.6.1.39
3.6.1.9
3.1.3.5
3.1.3.89
2.7.1.21
Thymidine
2.4.2.6
2.4.2.4
Thymine
2-Deoxy-D-ribose-1P
Pseudouridine-5'-phosphate
4.2.1.70
2.7.1.83
Pseudouridine
1.3.1.1
1.3.1.2
Dihydrouracil
3.5.2.2
3-Ureido-propionate
3.5.1.6
β-Alanine
β-Alanine metabolism
1.17.99.4
Barbiturate
3.5.2.1
3-Oxo-3-ureido-propanoate
3.5.1.95
Malonate
Urea
Ureidoacrylate
3.5.1.110
Carbamate
1.14.99.46
(Z)-3-Ureidoacrylate-peracid
3.5.1.110
(Z)-3-Peroxy-aminoacrylate
RutC
Aminoacrylate
RutD
Malonate-semialdehyde
YdfG
RutE
3-Hydroxy-propionate
5-Methylbarbiturate
1.17.99.4
3.5.2.1
3-Oxo-3-ureido-isobutyrate
3.5.1.95
Methylmalonate
Urea
1.3.1.1
1.3.1.2
(R)-Dihydro-thymine
3.5.2.2
(R)-3-Ureido-isobutyrate
3.5.1.6
(R)-3-Amino-isobutyrate
Valine, leucine and isoleucine metabolism
00240 1/9/19
(c) Kanehisa Laboratories

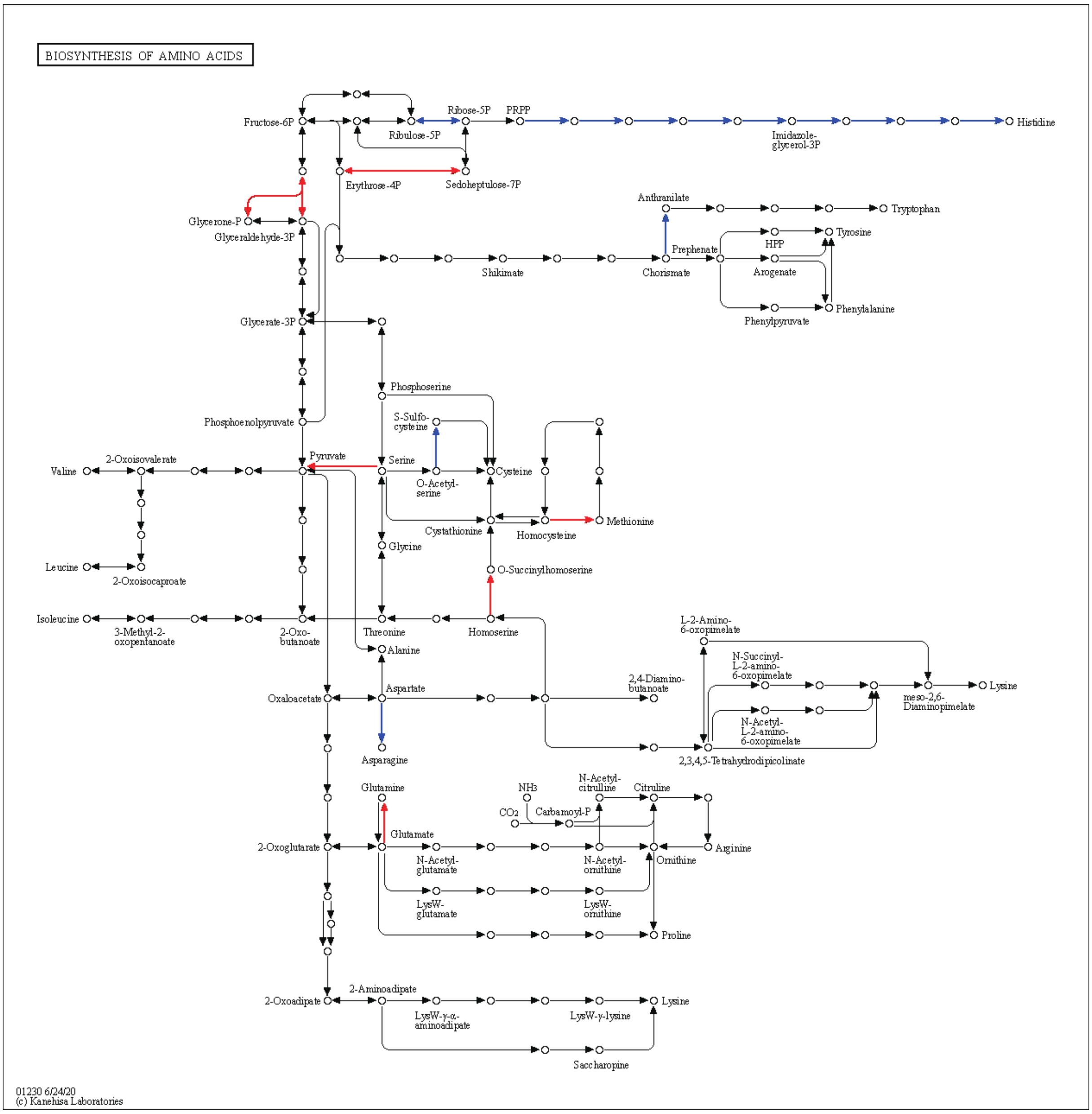
BIOSYNTHESIS OF AMINO ACIDS
Fructose-6P
Ribose-5P
PRPP
Ribulose-5P
Imidazole-
glycerol-3P
Histidine
Erythrose-4P
Sedoheptulose-7P
Glycerone-P
Glyceraldehyde-3P
Anthranilate
Tryptophan
Prephenate
HPP
Tyrosine
Shikimate
Chorismate
Arogenate
Phenylalanine
Phenylpyruvate
Glycerate-3P
Phosphoserine
Phosphoenolpyruvate
S-Sulfo-
cysteine
2-Oxoisovalerate
Pyruvate
Serine
Valine
O-Acetyl-
serine
Cysteine
Cystathionine
Homocysteine
Methionine
Glycine
Leucine
2-Oxoisocaproate
O-Succinylhomoserine
Isoleucine
3-Methyl-2-
oxopentanoate
2-Oxo-
butanoate
Threonine
Homoserine
L-2-Amino-
6-oxopimelate
Alanine
N-Succinyl-
L-2-amino-
6-oxopimelate
2,4-Diamino-
butanoate
Aspartate
Oxaloacetate
meso-2,6-
Diaminopimelate
Lysine
N-Acetyl-
L-2-amino-
6-oxopimelate
Asparagine
2,3,4,5-Tetrahydrodipicolinate
Glutamine
NH3
N-Acetyl-
citrulline
Citruline
CO2
Carbamoyl-P
Glutamate
2-Oxoglutarate
N-Acetyl-
glutamate
N-Acetyl-
ornithine
Ornithine
Arginine
LysW-
glutamate
LysW-
ornithine
Proline
2-Aminoadipate
2-Oxoadipate
LysW-γ-α-
aminoadipate
LysW-γ-lysine
Lysine
Saccharopine
01230 6/24/20
(c) Kanehisa Laboratories

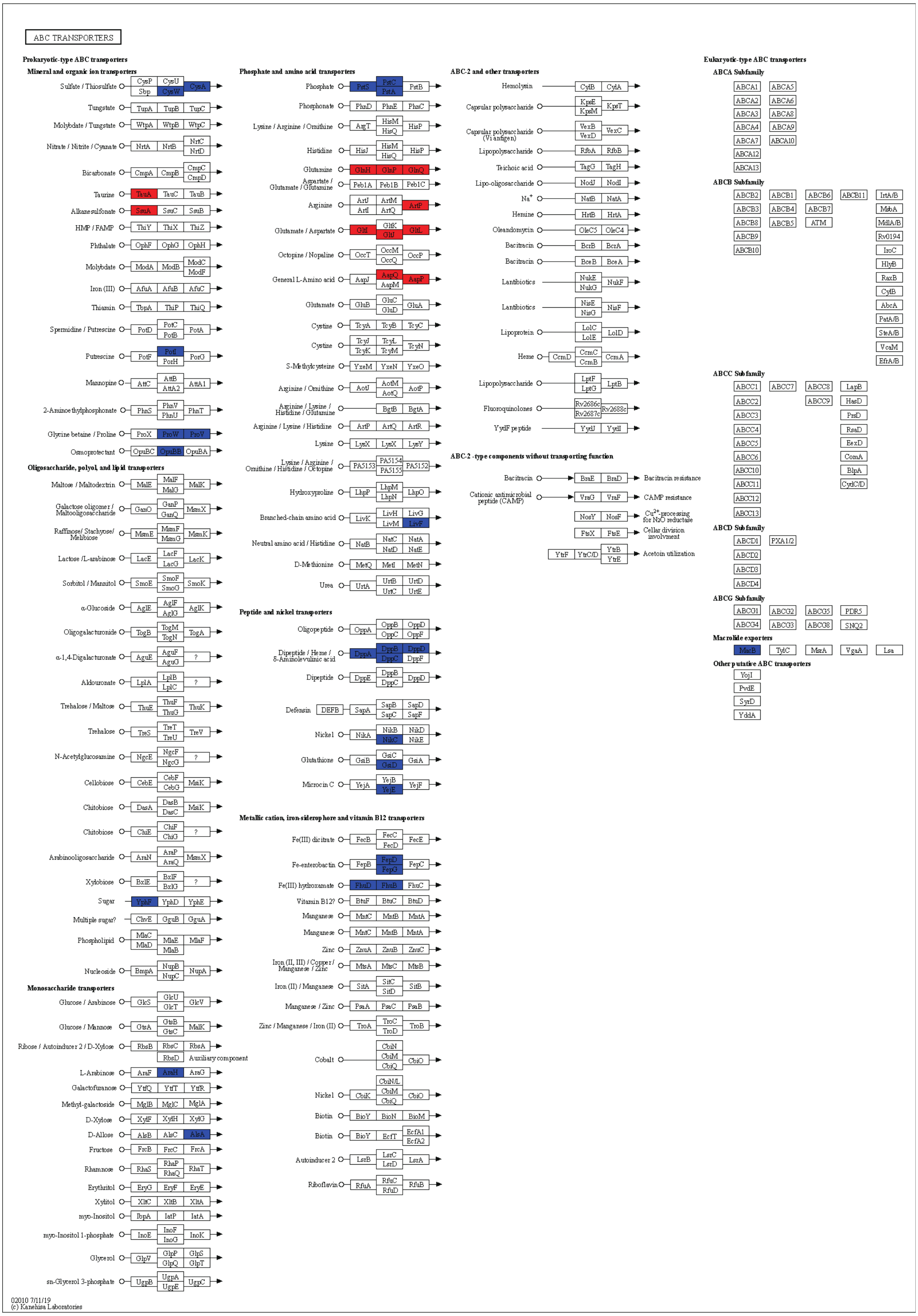
ABC TRANSPORTERS
Prokaryotic-type ABC transporters
Mineral and organic ion transporters
Sulfate / Thiosulfate CysP Sbp CysU CysW CysA
Tungstate TupA TupB TupC
Molybdate / Tungstate WtpA WtpB WtpC
Nitrate / Nitrite / Cyanate NrtA NrtB NrtC NrtD
Bicarbonate CmpA CmpB CmpC CmpD
Taurine TauA TauC TauB
Alkanesulfonate SsuA SsuC SsuB
HMP / FAMP ThiY ThiX ThiZ
Phthalate OphF OphG OphH
Molybdate ModA ModB ModC ModF
Iron (III) AfuA AfuB AfuC
Thiamin TbpA ThiP ThiQ
Spermidine / Putrescine PotD PotC PotB PotA
Putrescine PotF PotI PotH PotG
Mannopine AttC AttB AttA2 AttA1
2-Aminoethylphosphonate PhnS PhnV PhnU PhnT
Glycine betaine / Proline ProX ProW ProV
Osmoprotectant OpuBC OpuBB OpuBA
Oligosaccharide, polyol, and lipid transporters
Maltose / Maltodextrin MalE MalF MalG MalK
Galactose oligomer / Maltooligosaccharide GanO GanP GanQ MsmX
Raffinose/ Stachyose/ Melibiose MsmE MsmF MsmG MsmK
Lactose /L-arabinose LacE LacF LacG LacK
Sorbitol / Mannitol SmoE SmoF SmoG SmoK
α-Glucoside AglE AglF AglG AglK
Oligogalacturonide TogB TogM TogN TogA
α-1,4-Digalacturonate AguE AguF AguG ?
Aldouronate LplA LplB LplC ?
Trehalose / Maltose ThuE ThuF ThuG ThuK
Trehalose TreS TreT TreU TreV
N-Acetylglucosamine NgcE NgcF NgcG ?
Cellobiose CebE CebF CebG MsiK
Chitobiose DasA DasB DasC MsiK
Chitobiose ChiE ChiF ChiG ?
Arabinooligosaccharide AraN AraP AraQ MsmX
Xylobiose BxlE BxlF BxlG ?
Sugar YphF YphD YphE
Multiple sugar? ChvE GguB GguA
Phospholipid MlaC MlaD MlaE MlaB MlaF
Nucleoside BmpA NupB NupC NupA
Monosaccharide transporters
Glucose / Arabinose GlcS GlcU GlcT GlcV
Glucose / Mannose GtsA GtsB GtsC MalK
Ribose / Autoinducer 2 / D-Xylose RbsB RbsC RbsA RbsD Auxiliary component
L-Arabinose AraF AraH AraG
Galactofuranose YtfQ YtfT YtfR
Methyl-galactoside MglB MglC MglA
D-Xylose XylF XylH XylG
D-Allose AlsB AlsC AlsA
Fructose FrcB FrcC FrcA
Rhamnose RhaS RhaP RhaQ RhaT
Erythritol EryG EryF EryE
Xylitol XltC XltB XltA
myo-Inositol IbpA IatP IatA
myo-Inositol 1-phosphate InoE InoF InoG InoK
Glycerol GlpV GlpP GlpQ GlpS GlpT
sn-Glycerol 3-phosphate UgpB UgpA UgpE UgpC
Phosphate and amino acid transporters
Phosphate PstS PstC PstA PstB
Phosphonate PhnD PhnE PhnC
Lysine / Arginine / Ornithine ArgT HisM HisQ HisP
Histidine HisJ HisM HisQ HisP
Glutamine GlnH GlnP GlnQ
Aspartate / Glutamate / Glutamine PeblA PeblB PeblC
Arginine ArtJ ArtI ArtM ArtQ ArtP
Glutamate / Aspartate GltI GltK GltJ GltL
Octopine / Nopaline OccT OccM OccQ OccP
General L-Amino acid AapJ AapQ AapM AapP
Glutamate GluB GluC GluD GluA
Cystine TcyA TcyB TcyC
Cystine TcyJ TcyK TcyL TcyM TcyN
S-Methylcysteine YxeM YxeN YxeO
Arginine / Ornithine AotJ AotM AotQ AotP
Arginine / Lysine / Histidine / Glutamine BgtB BgtA
Arginine / Lysine / Histidine ArtP ArtQ ArtR
Lysine LysX LysY LysY
Lysine / Arginine / Ornithine / Histidine / Octopine PA5153 PA5154 PA5155 PA5152
Hydroxyproline LhpP LhpM LhpN LhpO
Branched-chain amino acid LivK LivH LivM LivG LivF
Neutral amino acid / Histidine NatB NatC NatD NatA NatE
D-Methionine MetQ MetI MetN
Urea UrtA UrtB UrtC UrtD UrtE
Peptide and nickel transporters
Oligopeptide OppA OppB OppC OppD OppF
Dipeptide / Heme / δ-Aminolevulinic acid DppA DppB DppC DppD DppF
Dipeptide DppE DppB DppC DppD
Defensin DEFB SapA SapB SapC SapD SapF
Nickel NikA NikB NikC NikD NikE
Glutathione GsiB GsiC GsiD GsiA
Microcin C YejA YejB YejE YejF
Metallic cation, iron-siderophore and vitamin B12 transporters
Fe(III) dicitrate FecB FecC FecD FecE
Fe-enterobactin FepB FepD FepG FepC
Fe(III) hydroxamate FhuD FhuB FhuC
Vitamin B12? BtuF BtuC BtuD
Manganese MntC MntB MntA
Manganese MntC MntB MntA
Zinc ZnuA ZnuB ZnuC
Iron (II, III) / Copper / Manganese / Zinc MtsA MtsC MtsB
Iron (II) / Manganese SitA SitC SitD SitB
Manganese / Zinc PsaA PsaC PsaB
Zinc / Manganese / Iron (II) TroA TroC TroD TroB
Cobalt CbiN CbiM CbiQ CbiO
Nickel CbiK CbiN/L CbiM CbiQ CbiO
Biotin BioY BioN BioM
Biotin BioY EcfT EcfA1 EcfA2
Autoinducer 2 LsrB LsrC LsrD LsrA
Riboflavin RfuA RfuC RfuD RfuB
ABC-2 and other transporters
Hemolysin CylB CylA
Capsular polysaccharide KpsE KpsM KpsT
Capsular polysaccharide (Vi antigen) VexB VexD VexC
Lipopolysaccharide RfbA RfbB
Teichoic acid TagG TagH
Lipo-oligosaccharide NodJ NodI
Na+ NatB NatA
Hemine HrtB HrtA
Oleandomycin OleC5 OleC4
Bacitracin BcrB BcrA
Bacitracin BceB BceA
Lantibiotics NukE NukG NukF
Lantibiotics NisE NisG NisF
Lipoprotein LolC LolE LolD
Heme CcmD CcmC CcmB CcmA
Lipopolysaccharide LptF LptG LptB
Fluoroquinolones Rv2686c Rv2687c Rv2688c
YydF peptide YydJ YydI
ABC-2 -type components without transporting function
Bacitracin BraE BraD Bacitracin resistance
Cationic antimicrobial peptide (CAMP) VraG VraF CAMP resistance
NosY NosF Cu2+-processing for N2O reductase
FtsX FtsE Cellar division involvment
YtrF YtrC/D YtrB YtrE Acetoin utilization
Eukaryotic-type ABC transporters
ABCA Subfamily
ABCA1 ABCA5 ABCA2 ABCA6 ABCA3 ABCA8 ABCA4 ABCA9 ABCA7 ABCA10 ABCA12 ABCA13
ABCB Subfamily
ABCB2 ABCB1 ABCB6 ABCB11 IrtA/B ABCB3 ABCB4 ABCB7 MsbA ABCB8 ABCB5 ATM MdlA/B ABCB9 Rv0194 ABCB10 IrtC HlyB RaxB CydB AbcA PstA/B SteA/B VcaM EfrA/B
ABCC Subfamily
ABCC1 ABCC7 ABCC8 LapB ABCC2 ABCC9 HasD ABCC3 PrsD ABCC4 RsaD ABCC5 EexD ABCC6 ComA ABCC10 BlpA ABCC11 CydC/D ABCC12 ABCC13
ABCD Subfamily
ABCD1 PXA1/2 ABCD2 ABCD3 ABCD4
ABCG Subfamily
ABCG1 ABCG2 ABCG5 PDR5 ABCG4 ABCG3 ABCG8 SNQ2
Macrolide exporters
MacB TylC MsrA VgaA Lsa
Other putative ABC transporters
YojI PvdE SyrD YddA
02010 7/11/19
(c) Kanehisa Laboratories

Supplementary Fig. S11 Representative metabolic pathway maps at 24 h from KEGG mapper. Red indicates upregulation and blue indicates downregulation of each gene.

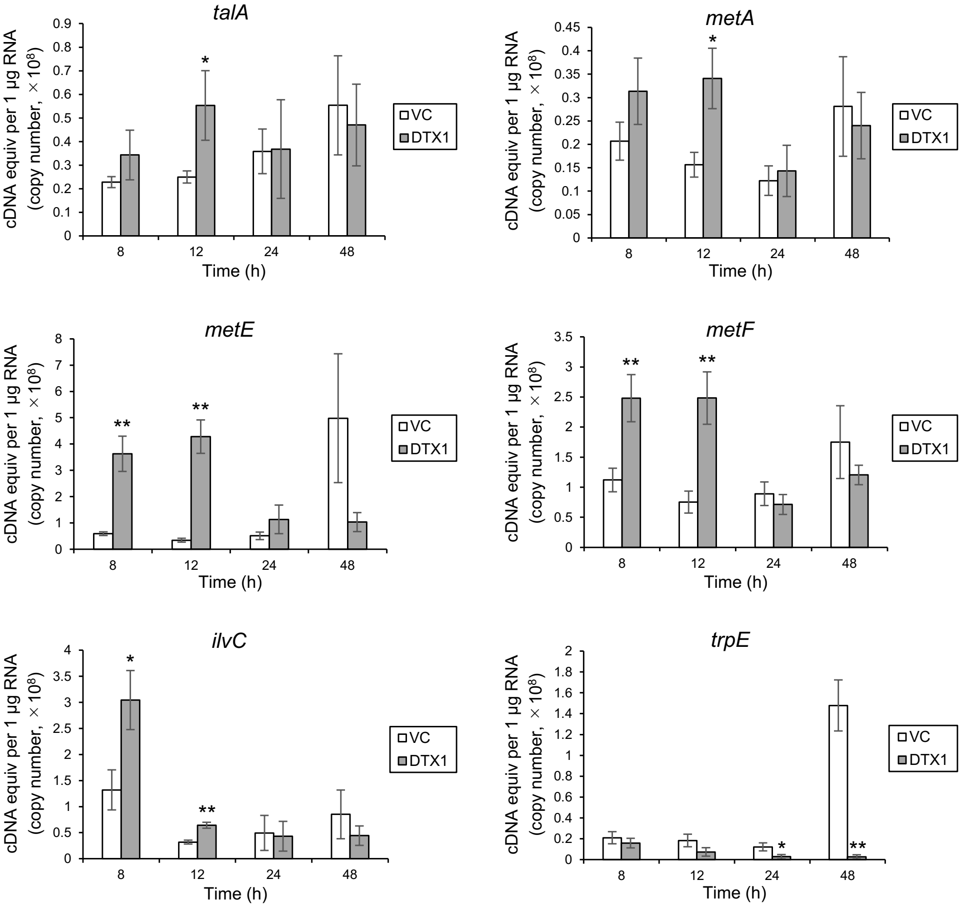

Supplementary Fig. S12 Temporal expression levels of several genes involved in the pentose phosphate pathway and the biosynthesis of methionine and other amino acids. The mRNA expression levels of *talA*, *metA*, *metE*, *metF*, *ilvC*, and *trpE* were determined by qRT-PCR using standard curve based on the copy number of plasmids. Error bars indicate the standard deviation calculated from three biological replicates. The asterisks denote significant differences according to Student's *t*-test compared with VC: $*P < 0.05$, $**P < 0.01$.

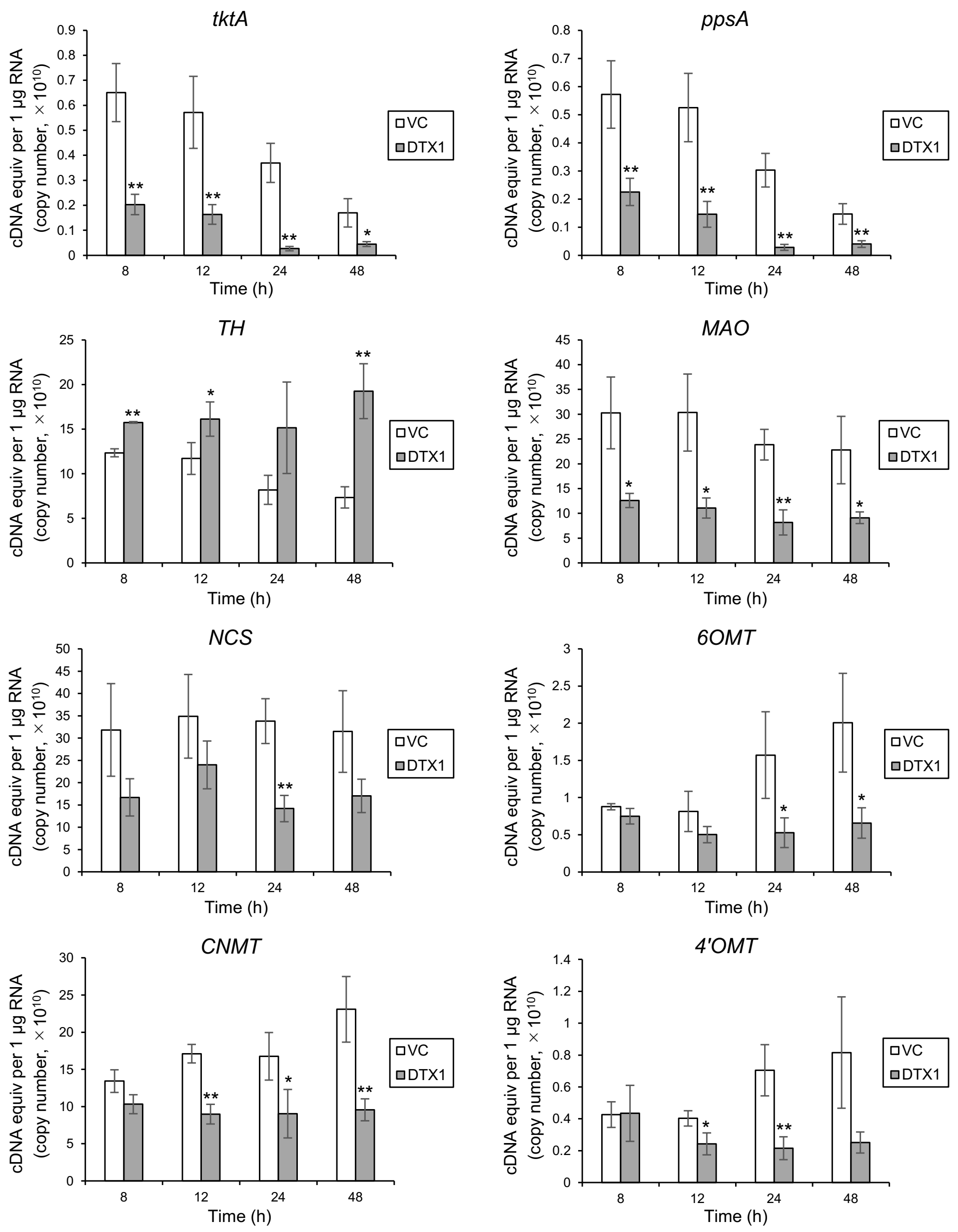

Supplementary Fig. S13 Temporal expression levels of exogenous genes involved in the biosynthesis of reticuline. The mRNA expression levels of *tktA*, *ppsA*, *TH*, *MAO*, *NCS*, *6OMT*, *CNMT*, and *4'OMT* were determined by qRT-PCR using standard curve based on the copy number of plasmids. Error bars indicate the standard deviation calculated from three biological replicates. The asterisks denote significant differences

according to Student's *t*-test compared with VC: *P < 0.05, **P < 0.01.

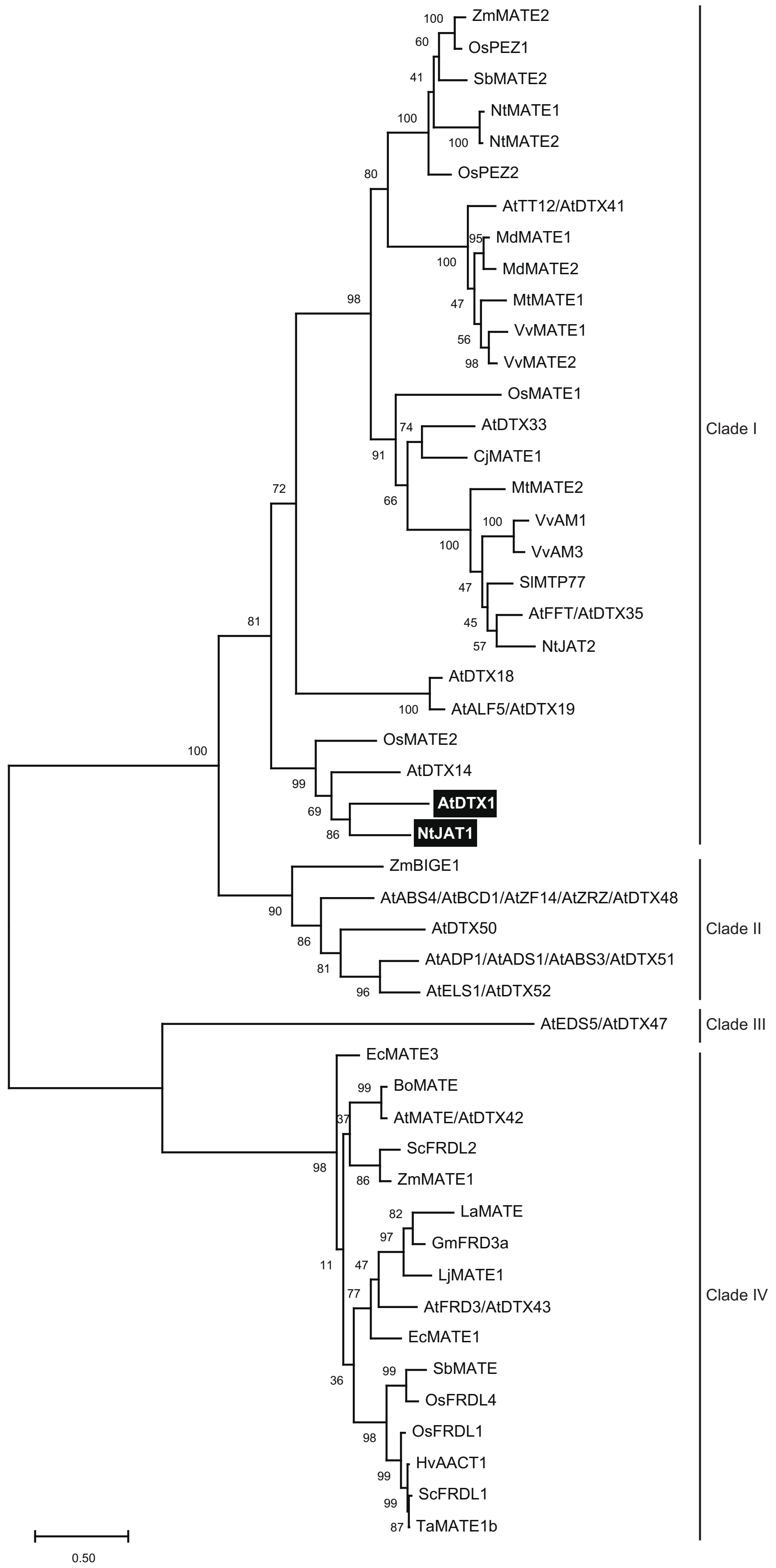
ZmMATE2
OsPEZ1
SbMATE2
NtMATE1
NtMATE2
OsPEZ2
AtTT12/AtDTX41
MdMATE1
MdMATE2
MtMATE1
VvMATE1
VvMATE2
OsMATE1
AtDTX33
CjMATE1
MtMATE2
VvAM1
VvAM3
SlMTP77
AtFFT/AtDTX35
NtJAT2
AtDTX18
AtALF5/AtDTX19
OsMATE2
AtDTX14
AtDTX1
NtJAT1
ZmBIGE1
AtABS4/AtBCD1/AtZF14/AtZRZ/AtDTX48
AtDTX50
AtADP1/AtADS1/AtABS3/AtDTX51
AtELS1/AtDTX52
AtEDS5/AtDTX47
EcMATE3
BoMATE
AtMATE/AtDTX42
ScFRDL2
ZmMATE1
LaMATE
GmFRD3a
LjMATE1
AtFRD3/AtDTX43
EcMATE1
SbMATE
OsFRDL4
OsFRDL1
HvAACT1
ScFRDL1
TaMATE1b
Clade I
Clade II
Clade III
Clade IV
0.50

Supplementary Fig. S14 Phylogenetic relationship of plant MATE family members. Plant MATE transporter sequences were aligned using ClustalW and subjected to phylogenetic analysis conducted using the MEGAX software (Kumar et al., 2018) and the Maximum Likelihood method and Le_Gascuel_2008 model (Le and Gascuel, 2008) with 1,000 bootstraps. The numbers on the branches represent bootstrap values. The scale bar shows the number of amino acid substitutions per site. Many proteins are indicated belonging to clade I transport secondary metabolites, such as cyanidin-3-*O*-glucoside, epicatechin 3′-*O*-glucoside, apigenin 7-*O*-glucoside, coumarolyagmatine, nicotine, berberine, palmatine, dhurrin, and protocatechuic acid. Some MATE transporters of clade I transport xenobiotics are indicated. MATE transporters of clade II are related to morphogenesis, disease resistance, leaf senescence, and ABA transport. AtEDS5/AtDTX47 (clade III) is involved in disease resistance *via* salicylic acid transport. Almost all clade IV proteins transport citrate and are implicated in Al^{3+} detoxification or Fe translocation.

Members of *Arabidopsis* MATE include the following: AtTT12/AtDTX41, At3g59030; AtDTX33, At1G47530; AtFFT/AtDTX35, At4g25640; AtDTX18, At3g23550; AtALF5/AtDTX19, At3g23560; AtDTX14, At1g71140; AtDTX1, At2g04070; AtABS4/AtBCD1/AtZF14/AtZRZ/AtDTX48, At1g58340; AtDTX50, At5g52050;

AtADP1/AtADS1/AtABS3/AtDTX51, At4g29140; AtELS1/AtDTX52, At5g19700; AtEDS5/AtDTX47, At4g39030; AtMATE/AtDTX42, At1g51340; AtFRD3/AtDTX43, At3g08040. Members of the MATE family in other plant species—and their accession numbers—include the following: BoMATE (*Brassica oleracea*), KF031944; CjMATE1 (*Coptis japonica*), BAX73926; EcMATE1 (*Eucalyptus camaldulensis*), BAM68465; EcMATE3, BAM68467; GmFRD3a (*Glycine max*), ACE89001; HvAACT1 (barley), BAF75822; LaMATE (*Lupinus albus*), AAW30732; LjMATE1 (*Lotus japonicus*), BAN59993; MdMATE1 (*Malus domestica*), ADO22710; MdMATE2, ADO22712; MtMATE1 (*Medicago truncatula*), ACX37118; MtMATE2, ADV04045; NtJAT1 (*Nicotiana tabacum*), CAQ51477; NtJAT2, BAP40098; NtMATE1, BAF47751; NtMATE2, BAF47752; OsFRDL1 (*Oryza sativa*), BAG95121; OsFRDL4, BAL41687; OsMATE1, Os03g08900; OsMATE2, Os05g48040; OsPEZ1, AK243209; OsPEZ2, Os03g0572900; SbMATE (*Sorghum bicolor*), ABS89149; SbMATE2, Sobic.001G012600; ScFRDL1 (rye), BAJ61741; ScFRDL2, BAJ61742; SlMTP77 (tomato), AAQ55183; TaMATE1b (*Triticum aestivum*), AFZ61900; VvAM1 (*Vitis vinifera*), ACN91542; VvAM3, ACN88706; VvMATE1, XP_002282907; VvMATE2, XP_002282932; ZmBIGE1 (*Zea mays*), KT310084; ZmMATE1, ACM47309; ZmMATE2, ACZ55931.

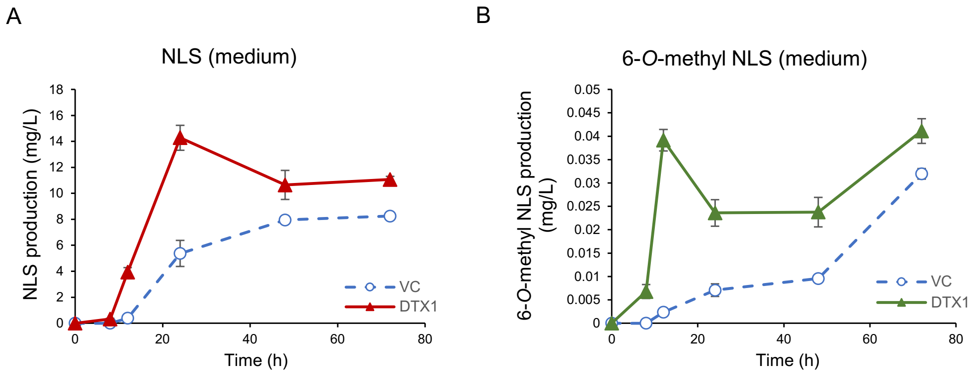

Supplementary Fig. S15 Accumulation of intermediates in *E. coli* cultured medium.

The time-dependent accumulation of (A) (*S*)-norlaudanosoline (NLS) and (B) (*S*)-6-*O*-methylnorlaudanosoline (6-*O*-methyl NLS) in the medium was determined by UPLC-MS.

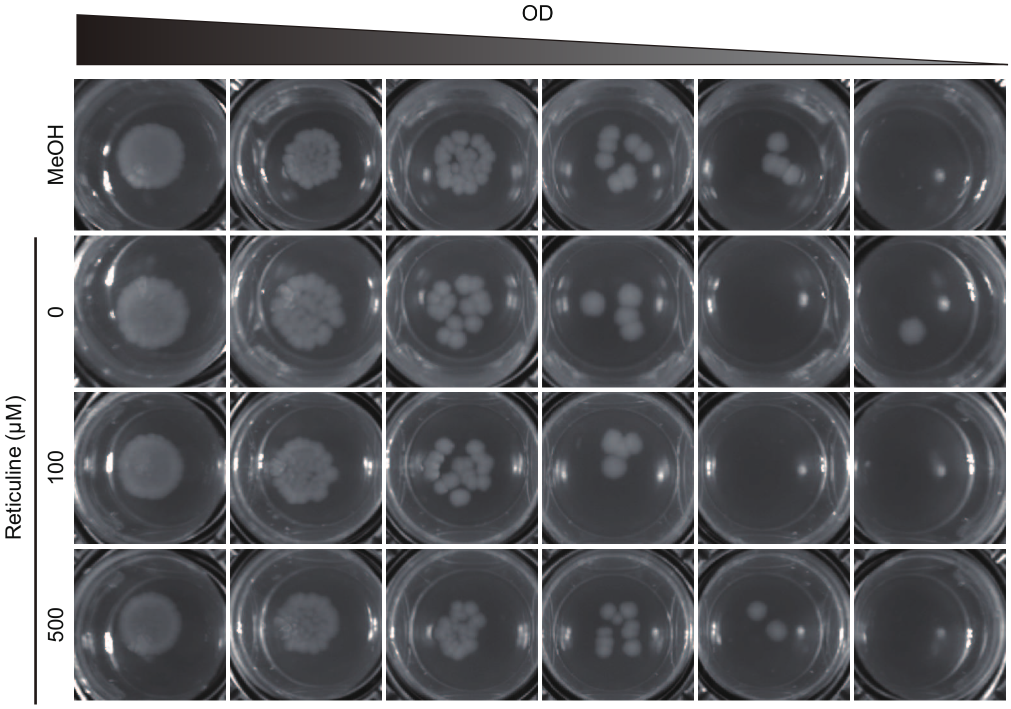

Supplementary Fig. S16 Growth of *E. coli* in LB medium containing reticuline. *E. coli* BL21(DE3) harboring pCOLADuet-1 vector were precultured in LB medium at 37 °C while shaking at 200 rpm. When the OD_{600} of the cultures reached 1.0, they were diluted to OD_{600} = 0.001. Serially diluted samples (5 μL each) were spotted onto a half-strength LB plate containing either 0, 100, or 500 μM reticuline and incubated for 36 h at 25°C; 5% (v/v) MeOH was used as the control.

Supplementary Table S1. The number of differentially expressed genes and associated KEGG pathway terms in AtDTX1-expressing cells

			Time point (h)				
**Metabolism**		Reg.	0	8	12	24	48
**Carbohydrate metabolism**	00010 Glycolysis / Gluconeogenesis	up	0	2	8	4	1
		down	0	0	2	4	3
	00020 Citrate cycle (TCA cycle)	up	0	1	3	1	0
		down	1	0	1	1	5
	00030 Pentose phosphate pathway	up	0	0	6	2	1
		down	0	1	0	3	1
	00040 Pentose and glucuronate interconversions	up	0	0	2	2	2
		down	0	2	0	3	1
	00051 Fructose and mannose metabolism	up	1	0	5	3	1
		down	1	1	3	6	11
	00052 Galactose metabolism	up	0	4	3	3	0
		down	1	0	1	3	8
	00053 Ascorbate and aldarate metabolism	up	0	0	0	0	0
		down	0	1	0	3	1
	00500 Starch and sucrose metabolism	up	0	0	5	2	2
		down	0	0	0	1	3
	00520 Amino sugar and nucleotide sugar metabolism	up	1	0	2	4	2
		down	1	0	5	5	11
	00620 Pyruvate metabolism	up	0	1	5	3	2
		down	1	0	1	2	0
	00630 Glyoxylate and dicarboxylate metabolism	up	0	3	5	1	3
		down	1	0	4	3	0
	00640 Propanoate metabolism	up	0	1	1	1	2
		down	0	1	1	6	2
	00650 Butanoate metabolism	up	0	3	4	1	1
		down	0	1	1	4	3
	00660 C5-Branched dibasic acid metabolism	up	0	1	2	0	0
		down	0	0	0	0	4
	00562 Inositol phosphate metabolism	up	0	0	0	0	0
		down	0	0	0	0	1

**Energy metabolism**	00190 Oxidative phosphorylation	up	0	0	1	0	0
		down	0	0	0	1	5
	00680 Methane metabolism	up	0	1	4	1	2
		down	0	1	6	7	2
	00910 Nitrogen metabolism	up	0	1	1	2	3
		down	0	2	4	4	4
	00920 Sulfur metabolism	up	0	0	4	3	1
		down	4	0	0	2	16
**Lipid metabolism**	00061 Fatty acid biosynthesis	up	0	0	0	0	0
		down	0	0	0	0	1
	00071 Fatty acid degradation	up	0	0	2	0	1
		down	0	0	1	3	0
	00121 Secondary bile acid biosynthesis	up	0	0	1	0	0
		down	0	0	0	0	0
	00561 Glycerolipid metabolism	up	0	0	4	3	1
		down	0	0	1	1	2
	00564 Glycerophospholipid metabolism	up	0	0	1	1	1
		down	0	0	2	2	3
	00565 Ether lipid metabolism	up	0	0	0	0	0
		down	0	0	0	0	0
	00600 Sphingolipid metabolism	up	0	0	0	0	0
		down	1	0	0	1	2
	00590 Arachidonic acid metabolism	up	0	0	0	0	0
		down	0	0	0	0	0
	00592 alpha-Linolenic acid metabolism	up	0	0	0	0	0
		down	0	0	0	0	0
	01040 Biosynthesis of unsaturated fatty acids	up	0	0	1	0	2
		down	1	0	1	0	0
**Nucleotide metabolism**	00230 Purine metabolism	up	0	0	3	3	6
		down	1	1	4	8	7
	00240 Pyrimidine metabolism	up	0	1	4	14	4
		down	1	0	4	1	4
**Amino acid metabolism**	00250 Alanine, aspartate and glutamate metabolism	up	0	1	1	4	4
		down	0	0	2	6	2
		up	0	5	5	1	1

	00260 Glycine, serine and threonine metabolism	down	0	0	1	3	7
	00270 Cysteine and methionine metabolism	up	0	2	5	5	4
		down	0	0	0	2	3
	00280 Valine, leucine and isoleucine degradation	up	0	0	0	0	0
		down	0	0	0	3	0
	00290 Valine, leucine and isoleucine biosynthesis	up	0	2	3	0	0
		down	0	0	0	0	2
	00300 Lysine biosynthesis	up	0	0	1	0	0
		down	0	0	0	0	1
	00310 Lysine degradation	up	0	0	1	1	1
		down	0	0	0	3	2
	00220 Arginine biosynthesis	up	0	0	0	1	2
		down	0	0	2	0	1
	00330 Arginine and proline metabolism	up	0	0	1	3	3
		down	0	0	0	0	0
	00340 Histidine metabolism	up	0	0	0	0	0
		down	0	0	0	8	5
	00350 Tyrosine metabolism	up	0	0	2	0	1
		down	0	0	1	3	1
	00360 Phenylalanine metabolism	up	0	1	0	2	4
		down	0	0	0	4	2
	00380 Tryptophan metabolism	up	0	0	1	1	2
		down	0	0	0	1	2
	00400 Phenylalanine, tyrosine and tryptophan biosynthesis	up	0	0	1	0	0
		down	0	0	0	2	8
**Metabolism of other amino acids**	00410 beta-Alanine metabolism	up	0	1	0	0	0
		down	0	0	2	3	3
	00430 Taurine and hypotaurine metabolism	up	0	0	1	0	0
		down	0	0	0	0	1
	00440 Phosphonate and phosphinate metabolism	up	0	0	0	0	0
		down	0	1	0	0	0
	00450 Selenocompound metabolism	up	0	2	4	2	1
		down	1	1	0	0	4
	00460 Cyanoamino acid metabolism	up	0	0	0	0	0

		down	0	0	0	1	0
	00471 D-Glutamine and D-glutamate metabolism	up	0	0	0	0	0
		down	0	0	1	0	1
	00473 D-Alanine metabolism	up	0	0	0	0	0
		down	0	0	0	0	0
	00480 Glutathione metabolism	up	0	2	4	3	3
		down	0	0	0	0	0
**Glycan biosynthesis and metabolism**	00540 Lipopolysaccharide biosynthesis	up	0	0	0	2	0
		down	0	0	3	3	6
	00541 O-Antigen nucleotide sugar biosynthesis	up	1	0	0	1	0
		down	1	0	3	3	2
	00550 Peptidoglycan biosynthesis	up	0	0	0	0	0
		down	0	0	0	1	8
	00511 Other glycan degradation	up	0	0	0	0	0
		down	1	0	0	1	1
**Metabolism of cofactors and vitamins**	00730 Thiamine metabolism	up	0	0	0	0	1
		down	0	0	0	4	3
	00740 Riboflavin metabolism	up	0	0	0	1	1
		down	0	0	0	0	1
	00750 Vitamin B6 metabolism	up	0	0	0	0	1
		down	0	0	0	0	1
	00760 Nicotinate and nicotinamide metabolism	up	0	1	1	2	4
		down	0	0	0	2	0
	00770 Pantothenate and CoA biosynthesis	up	0	3	2	0	0
		down	0	0	2	1	4
	00780 Biotin metabolism	up	0	0	0	0	1
		down	0	1	2	2	0
	00785 Lipoic acid metabolism	up	0	0	0	0	0
		down	0	0	0	0	0
	00790 Folate biosynthesis	up	0	0	4	3	0
		down	0	0	0	1	0
	00670 One carbon pool by folate	up	0	2	2	0	0
		down	0	0	1	2	1
	00860 Porphyrin and chlorophyll metabolism	up	0	0	0	1	0
		down	0	0	0	1	0

	00130 Ubiquinone and other terpenoid-quinone biosynthesis	up	0	0	1	2	2
		down	0	0	2	1	2
**Metabolism of terpenoids and polyketides**	00900 Terpenoid backbone biosynthesis	up	0	0	0	0	0
		down	0	0	0	0	2
	00903 Limonene and pinene degradation	up	0	0	0	0	0
		down	0	0	0	1	0
	00281 Geraniol degradation	up	0	0	1	1	0
		down	0	0	0	1	0
	00523 Polyketide sugar unit biosynthesis	up	0	0	0	0	0
		down	0	0	0	5	0
	01053 Biosynthesis of siderophore group nonribosomal peptides	up	0	0	0	0	2
		down	0	0	0	0	1
**Biosynthesis of other secondary metabolites**	00332 Carbapenem biosynthesis	up	0	0	0	0	0
		down	0	0	0	0	0
	00261 Monobactam biosynthesis	up	0	0	0	0	0
		down	1	0	0	0	2
	00521 Streptomycin biosynthesis	up	0	0	0	0	0
		down	0	0	0	0	1
	00525 Acarbose and validamycin biosynthesis	up	0	0	0	0	0
		down	0	0	0	0	0
	00401 Novobiocin biosynthesis	up	0	0	0	0	0
		down	0	0	0	1	1
**Xenobiotics biodegradation and metabolism**	00362 Benzoate degradation	up	0	0	0	0	0
		down	0	0	0	1	0
	00627 Aminobenzoate degradation	up	0	0	0	0	0
		down	0	1	1	0	0
	00364 Fluorobenzoate degradation	up	0	0	0	0	0
		down	0	0	0	0	0
	00625 Chloroalkane and chloroalkene degradation	up	0	0	2	0	1
		down	0	0	1	1	0
	00361 Chlorocyclohexane and chlorobenzene degradation	up	0	0	0	0	0
		down	0	0	0	0	0
	00623 Toluene degradation	up	0	0	0	0	0
		down	0	0	0	0	0
	00622 Xylene degradation	up	0	0	0	0	0

		down	0	0	0	0	0
	00633 Nitrotoluene degradation	up	0	0	1	1	1
		down	0	0	1	0	1
	00930 Caprolactam degradation	up	0	0	0	0	0
		down	0	0	0	1	0
	00621 Dioxin degradation	up	0	0	0	0	0
		down	0	0	0	0	0
	00626 Naphthalene degradation	up	0	0	2	0	1
		down	0	0	1	1	0

**Genetic Information Processing**		**(h)**	**0**	**8**	**12**	**24**	**48**
**Transcription**	03020 RNA polymerase	up	0	0	0	0	0
		down	0	0	0	0	0
**Translation**	03010 Ribosome	up	0	0	0	0	0
		down	0	0	23	1	1
	00970 Aminoacyl-tRNA biosynthesis	up	0	0	0	0	0
		down	0	0	0	0	0
**Folding, sorting and degradation**	03060 Protein export	up	0	0	0	0	0
		down	0	0	0	1	3
	04122 Sulfur relay system	up	0	0	3	0	1
		down	0	0	1	1	2
	03018 RNA degradation	up	0	0	0	0	0
		down	0	0	2	0	1
**Replication and repair**	03030 DNA replication	up	0	0	1	2	0
		down	0	0	1	1	3
	03410 Base excision repair	up	0	0	1	1	2
		down	0	0	1	1	3
	03420 Nucleotide excision repair	up	0	0	1	0	0
		down	0	0	0	1	0
	03430 Mismatch repair	up	0	0	0	2	0
		down	0	0	1	1	3
	03440 Homologous recombination	up	0	0	1	2	0
		down	0	0	3	0	5

**Environmental Information Processing**		**(h)**	**0**	**8**	**12**	**24**	**48**
**Membrane transport**	02010 ABC transporters	up	0	3	8	11	16

		down	1	10	12	25	30
	02060 Phosphotransferase system (PTS)	up	0	3	5	6	0
		down	0	0	2	2	13
	03070 Bacterial secretion system	up	0	0	0	0	0
		down	0	0	1	0	3
**Signal transduction**	02020 Two-component system	up	0	1	3	15	15
		down	1	5	11	23	15

**Cellular Processes**		**(h)**	**0**	**8**	**12**	**24**	**48**
**Cellular community - prokaryotes**	02024 Quorum sensing	up	0	0	0	2	3
		down	0	2	4	3	8
	02026 Biofilm formation - Escherichia coli	up	0	0	1	3	4
		down	0	1	1	3	4
**Cell motility**	02030 Bacterial chemotaxis	up	0	0	0	1	0
		down	0	1	1	8	3
	02040 Flagellar assembly	up	0	4	1	3	0
		down	0	0	2	0	11

**Human Diseases**		**(h)**	**0**	**8**	**12**	**24**	**48**
**Drug resistance: antimicrobial**	01501 beta-Lactam resistance	up	0	0	0	1	0
		down	0	0	1	0	3
	01502 Vancomycin resistance	up	0	0	0	1	1
		down	0	0	0	0	1
	01503 Cationic antimicrobial peptide (CAMP) resistance	up	0	0	0	3	3
		down	0	0	0	0	2

Reg, regulation.

Supplementary Table S2. Primer list usef for quantitative real-time PCR

Primer name	Oligonucleotide sequences (5' to 3')
*talA*_RT_Fw	CCTGGGAAGGAATTCGCG
*talA*_RT_Rv	GCTTGCGTGCCTGATACCAG
*metA*_RT_Fw	CGCAGATCAAACAGGTGCTG
*metA*_RT_Rv	AAGCGCATGAGGATGGAGAA
*metE*_RT_Fw	CCAGGGACAGGTGAAACTGC
*metE*_RT_Rv	AGCCAGTCAGAAGGCAGACG
*metF*_RT_Fw	TCAGAGCCTGGCAGAAGTCC
*metF*_RT_Rv	GAGTTCGCGCCATAGGTCAC
*ilvC*_RT_Fw	TGCTGGAATCGTCCTTCGTT
*ilvC*_RT_Rv	TTCGGTGATGGTTTCCCAAC
*trpE*_RT_Fw	CACCGCTTAACGTCCCCATA
*trpE*_RT_Rv	TCGCCGATCTCACCAAAGTT
*tktA*_RT_Fw	GATCCTCTCCCGTCAGAACCT
*tktA*_RT_Rv	CGGCAGTCAGTTTTTCGTAGG
*ppsA*_RT_Fw	GGGTAAGATTATCGCCGAAGG
*ppsA*_RT_Rv	CGGTCATGTCAGTAACCAGCA
*MAO*_RT_Fw	CATTTGCGTGGGCTTTGG
*MAO*_RT_Rv	ACGCCACTGATCCACAATCG
*NCS*_RT_Fw	GCGAACGCCTGATTTTTAATG
*NCS*_RT_Rv	TGCCTTCGACAGACCAAACTT
*DroTH*_RT_Fw	TGAATCCCAATCACAGGAACC
*DroTH*_RT_Rv	ATAGCTGCTTCGGCATCAGAG
*6OMT*_RT_Fw	GTGCATCCTTCACGACTGG
*6OMT*_RT_Rv	TGCATCATGGATGAGCTTCT
*CNMT*_RT_Fw	ACCACACATGAGATGGCTGA
*CNMT*_RT_Rv	CCCAGCAGGAAACACGTACT
*4'OMT*_RT_Fw	GGAAGGACACCCTGATCAAA
*4'OMT*_RT_Rv	TTCCTCCACCAACATCAACA

Legends for Supplementary Datasets

Supplementary Data S1. Summary of RNA-seq data.

Supplementary Data S2. Summary of genes induced or suppressed in AtDTX1-expressing cells, with |fold change| ≥ 2. Statistical analysis was performed using Fold-Change (FC). exactTest using edgeR was performed per comparison pair. Significant results were selected based on |FC| ≥ 2 and exactTest raw *P*-value < 0.05.

Supplementary Data S3. Gene Ontology analysis of significantly altered genes at 0 h (AtDTX1 vs. vector control). Statistical analysis was performed using Fold-Change (FC). exactTest using edgeR was performed per comparison pair. Significant results were selected based on |FC| ≥ 2 and exactTest raw *P*-value < 0.05.

Supplementary Data S4. Gene Ontology analysis of significantly altered genes at 8 h (AtDTX1 vs. vector control). Statistical analysis was performed using Fold-Change (FC); exactTest was performed using edgeR per comparison pair. The significant results were selected based on |FC| ≥ 2 and exactTest raw *P*-value < 0.05.

Supplementary Data S5. Gene Ontology analysis of significantly altered genes at 12 h (AtDTX1 vs. vector control). Statistical analysis was performed using Fold-Change (FC). exactTest was performed using edgeR per comparison pair. The significant results were selected based on $|FC| \geq 2$ and exactTest raw *P*-value < 0.05.

Supplementary Data S6. Gene Ontology analysis of significantly altered genes at 24 h (AtDTX1 vs. vector control). Statistical analysis was performed using Fold-Change (FC). exactTest was performed using edgeR per comparison pair. The significant results were selected based on $|FC| \geq 2$ and exactTest raw *P*-value < 0.05.

Supplementary Data S7. Gene Ontology analysis of significantly altered genes at 48 h (AtDTX1 vs. vector control). Statistical analysis was performed using Fold-Change (FC). exactTest was performed using edgeR per comparison pair. The significant results were selected based on $|FC| \geq 2$ and exactTest raw *P*-value < 0.05.

References

1. Kumar S., Stecher G., Li M., Knyaz C., Tamura K., 2018. MEGA X: molecular evolutionary genetics analysis across computing platforms. *Mol. Biol. Evol.* 35, 1547–1549. https://doi.org/10.1093/molbev/msy096.
2. Le S.Q., Gascuel O., 2008. An improved general amino acid replacement matrix. *Mol. Biol Evol.* 25(7), 1307–1320. https://doi.org/10.1093/molbev/msn067.
